# A risk-averse sustainable perishable food supply chain considering production and delivery times with real-world application

**DOI:** 10.1371/journal.pone.0308332

**Published:** 2024-09-26

**Authors:** Mahdieh Shakuri, Farnaz Barzinpour

**Affiliations:** School of Industrial Engineering, Iran University of Science & Technology, Narmak, Tehran, Iran; Wuhan Textile University, CHINA

## Abstract

In recent years, a relatively novel paradigm known as sustainable development has been introduced in response to concerns regarding the adverse impact of industrial activities on the environment and society. Managers in the food sector have been attempting to incorporate the principle of sustainable development in their supply chains owing to the paramount importance of social and environmental considerations in creating a competitive advantage for food products. To this end, we propose a multi-objective linear mathematical model considering the three dimensions of sustainability, i.e. economic, environmental, and social, to design a sustainable food supply chain. Given today’s volatile business environment, we employ a robust optimization model by incorporating Conditional Value-at-Risk into the configuration of two-stage stochastic programming to tackle uncertainty and take up a risk-averse strategy in supply chain design. The model aims to identify the optimal production and delivery times of the products, investigate the effects of their perishability characteristic on inventory decisions, and assess the financial and environmental advantages of transportation decisions to improve the sustainability of logistics operations. A novel version of fuzzy goal programming approach is applied to solve the proposed model. Next, the applicability of the proposed model and its solution method is verified based on computational experiments on a real-world case study of a processed food company. Lastly, conflicts between the sustainability aspects are examined, and several sensitivity analyses on risk-aversion parameters are performed to provide managerial insights for industry executives seeking to optimize their network concerning sustainability issues and well-performance under worst-case scenarios.

## 1. Introduction

A supply chain (SC) is a set of processes and flows that take place within and between different network components, including all parties directly or indirectly involved in fulfilling customers’ demands. The food supply chain (FSC) is perceived as critical infrastructure as it ensures the wellbeing and food security of customers and yields profit for the firms involved [1]. According to projections, the global food demand is expected to rise by 50% by 2030 [2]. The food industry has experienced enormous challenges in recent decades. The COVID-19 pandemic, for instance, has caused a worldwide spike in food demand over the last four years. Consequently, optimal decisions and efficient SC management are needed to respond to the continuously growing food demand [3]. Researchers and practitioners often classify SC decisions according to the length of the planning horizon into three categories: strategic, tactical, and operational [4]. Supply chain network design (SCND) comprises a set of crucial strategic decisions that determine the number, location, and capacity of facilities as well as the flow between them [5]. Through its influence on tactical and operational decisions, SCND significantly affects product quality, service level, material flow, customer satisfaction, and return on investment [6]. Typically, SCND in the food industry involves a high level of complexity due to the unique characteristics of foodstuff, such as perishability [7, 8]. Unlike many types of commodities, the demand for food is directly affected by the age and freshness of the products. Hence, failure to provide customers with high-quality, fresh food products often leads to a decrease in demand and thereby a dramatic decline in profitability [9]. Therefore, the perishable nature of food products imposes additional costs on the SC and requires continuous monitoring and replenishment of inventory levels in facilities to keep the inventory fresh and consumable [10].

Sustainability is among the significant issues to consider in the network design of FSCs. A SC is considered sustainable when the SC management policies and innovations of the organization are aligned with the principles of sustainable development. Achieving sustainable development requires balance between economic growth, environmental protection, and social welfare [11]. Sustainable Development Goals are a set of interconnected objectives proposed by the United Nations that call for emphasis on developing sustainable FSCs and effecting fundamental changes to improve the current food systems by 2030 [12]. The World Health Organization reports that FSCs release a significant amount of pollutants which are responsible for an estimated 20 to 30 percent of all environmental impacts [13].

Also, according to the Food and Agriculture Organization of the United Nations (FAO), over one-third of all food produced annually is wasted. So, food waste is the biggest threat to global food security, and reducing food waste is essential to achieving global food security and sustainable development [14]. On the other hand, risk management in the field of SCND has drawn increasing interest among scholars and industry practitioners [15]. There is a high degree of interdependence between network nodes in any type of SC. Therefore, the risks generated in one node often spread quickly to others and negatively impact their operational and sustainability performance [16]. The occurrence of risks is widely believed to reduce the profitability and efficiency of the SC. In the case of FSCs, risks may also accelerate the products’ deterioration rate, adversely affecting the environment and the firm’s reputation [17]. In this regard, effective risk management is essential to ensure sustainable operations [18]. SC risk refers to potential losses in terms of the objectives resulting from uncertainties in the features of the SC that can arise when a triggering event occurs [19]. In general, two types of risks can be associated with SCND problems: disruption risks and operational risks [20]. Disruption risks, including natural disasters (e.g. floods, earthquakes, and storms), human-made crises (e.g. wars and terrorist attacks), and technological malfunctions (e.g. equipment failure), can significantly undermine the performance of SCs. Operational risks, on the other hand, arise due to uncertainties in parameters such as supply, demand, transportation costs, etc. in dynamic business environments. To be precise, this type of risk is caused by the deviation of stochastic parameters from their expected values, leads to potential financial losses, and hampers the company’s efforts to meet its objectives [21, 22]. The risk-averse approach was developed to cope with uncertain parameters and guide managers when making decisions under uncertain conditions [23]. When addressing FSC problems, it is important to consider the above-mentioned concerns simultaneously.

To the best of our knowledge, the present study is among the first attempts to investigate a comprehensive risk-averse sustainable FSC design problem. In this regard, we model the risk aversion approach using the Conditional Value-at-Risk (CVaR), a well-known risk measure to tackle random outcomes, volatility, and the inherent uncertainties of SCs. The proposed multi-period multi-objective programming model considers the perishability of food products based on the production and delivery times of the products and their shelf-life. Also, by addressing three objective functions, the economic, environmental, and social pillars of sustainable development are taken into consideration. We use preemptive fuzzy goal programming (PFGP), a novel multi-objective method, to solve the model. Lastly, we test the applicability of the mathematical model through computational experiments conducted on a numerical example and provide a number of managerial insights into some of the model’s critical parameters.

The primary contributions, and novelty of this research can be summarized as follows:

- Developing a robust, sustainable, risk-averse FSC utilizing the coherent risk measure;
- Considering perishability in inventory decisions based on the production times, delivery times, and shelf-life of the products;
- Investigating product transportation decisions in terms of sustainability implications in FSC;
- Considering job creation and safety together as the social impacts in the FSC;
- Proposing an improved version of goal programming to solve the presented model;
- Providing a real case study in the processed food industry to validate the proposed model and solution approach.

The remainder of this paper is structured as follows: Section 2 presents the literature review of the subject. Section 3 describes the problem and its mathematical formulation. Section 4 presents the solution approach of the proposed model. Section 4 is devoted to the real-world case study, computational results and sensitivity analyses on some crucial parameters. Section 5 discuses some managerial insights and practical implications. Eventually, Section 6 provides conclusions and suggestions for future research.

## 2. Literature review

In this section, we review the relevant prior research from three different perspectives: (1) food supply chain network design (FSCND) problems, (2) sustainable SCND problems, and (3) risk-averse SCND problems involving risk measures and uncertainty. For the works reviewed in each subsection, we provide a brief summary and then mention the contributions of our study in that regard. Finally, at the end of this section, the details of the relevant prior research and the proposed study are summarized in Table 1 to clarify research gaps.

**Table 1:** Summary of the relevant prior research.

Authors	Year	Sustainability			Capacity planning	Mode selection	Perishability	Production & delivery	Risk-aversion	Uncertainty	Multi-period	Multi-product	Case study	Solution approach
		Economic	Environmental	Social										
Allaoui et al. [2]	2018	*	*	*		*					*	*	Agro-food	CPLEX
Aras & Bilge [27]	2018	*								*	*	*	Food	CPLEX
Rahimi & Ghezavati [46]	2018	*	*	*					*	*	*		–	Ε-constraint
Rahimi et al. [47]	2019	*	*	*					*	*	*	*	–	Ε-constraint
Mohammadi et al. [34]	2020	*	*	*		*	*				*	*	Processed food	Augmented ε-constraint
Mohebalizadehgashti et al. [28]	2020	*	*							*	*	*	Meat	Augmented ε-constraint
Patidar & Agrawal [29]	2020	*					*				*	*	Vegetable	LINGO
Yakavenka et al. [33]	2020	*	*	*		*							Fruit	Goal programming
Arabsheybani & Arshadi Khasmeh [31]	2021	*								*	*	*	Flavor	Ε-constraint
Gholami-Zanjani, et al. [13]	2021	*	*			*	*			*	*	*	Meat	Lexicographic weighted Tchebycheff
Gholami-Zanjani et al. [31]	2021	*					*			*	*		Food	CPLEX
Jouzdani & Govindan [35]	2021	*	*	*		*	*			*	*	*	Dairy	Multi-choice goal programming
Goodarzian et al. [36]	2022	*	*				*				*	*	Fruit	Augmented ε-constraint, Hybrid meta-heuristic algorithm
Kalantari & Hosseininezhad [12]	2022	*	*	*			*			*	*	*	Food	Cross-entropy
Goodarzian et al. [39]	2023	*	*	*							*		Fruit	Ε-constraint, Meta-heuristic algorithm
Mohammadi et al. [40]	2023	*	*	*			*					*	Processed food	PFGP
Lotfi et al. [49]	2023	*							*	*			–	GAMS
Lotfi et al. [50]	2024	*	*						*	*			Healthcare industry	GAMS
**This study**		*	*	*	*	*	*	*	*	*	*	*	**Processed food**	**PFGP**

### 2.1. Food supply chain network design

Due to the growing importance of food, there has been an increasing number of studies on the subject of FSCND in recent years. Mohammed and Wang [24] proposed a multi-objective model to design and plan a meat SC. The researchers used three different methods to solve the multi-objective model and, in the end, opted to use the Technique for Order of Preference by Similarity to Ideal Solution (TOPSIS) to obtain the optimal solution from the Pareto frontier. Mohammed et al. [25] expanded the aforementioned approach by presenting a multi-objective model that used stochastic programming to deal with uncertainty. The authors employed the monitoring system known as Radio-Frequency Identification (RFID) to satisfy the objectives of their model. Mohammed and Wang [26] presented another mathematical model to improve the distribution system of their earlier meat SC model. Aras and Bilge [27] presented a mixed-integer linear programming model to design a FSC in Turkey. The authors utilized the minimax regret approach to deal with uncertain demand. Mohebalizadehgashti et al. [28] formulated a meat SC with the objective of minimizing the total cost and CO2 emission, as well as maximizing the capacity utilization rate of the facilities in the network. Patidar and Agrawal [29] introduced a mixed-integer non-linear programming formulation to optimize the total cost of an agricultural SC. The results indicated that the expenses incurred by farm-to-market transportation were responsible for 85% of the total SC cost. To mitigate this defect, they proposed that the farmers be classified into clusters such that their crops would be aggregated in cluster centers and transported to the market from there. Gholami-Zanjani et al. [30] developed a two-stage scenario-based model with uncertain demand and disruptions caused by an epidemic. The researchers incorporated a number of resilience strategies into the core model to design a resilient FSC. Arabsheybani and Arshadi Khasmeh [31] proposed a robust bi-objective model to design a resilient FSC with the objectives being to maximize the total profit and total resilience score. Relying on four criteria to enhance the resilience of the SC (robustness, agility, leanness, and flexibility). Gholami-Zanjani et al. [13] developed a new framework to configure a resilient green meat SC. The authors implemented two resilience strategies to hedge the network against disruptions and solved the model by applying the sample average approximation and lexicographic weighted Tchebycheff methods.

### 2.2. Sustainable SCND

The concept of sustainability has gained popularity in the last few years since it demonstrates that a SC’s competitive advantages cannot only be achieved through optimizing its economic aspects [32]. Sustainability is more emphasized in some industries, such as the food industry, due to the specific characteristics of their products. In this subsection, we survey some of the studies on sustainable SCND problems.

Allaoui et al. [2] came up with a hybrid two-stage methodology to optimize a sustainable SC in which sustainable suppliers are selected using the Analytic Hierarchy Process (AHP). Yakavenka et al. [33] presented a distribution network design problem for perishable food products considering the three dimensions of sustainability. The proposed model’s objectives were to minimize the total cost, total emission of transportation modes, and transportation times. Mohammadi et al. [34] developed a sustainable multi-objective model for a processed food company. The decisions made by proposed model included facility location, aggregating the flow of materials between facilities, and selecting the type of product delivery (either directly from plants or indirectly from distribution centers). Jouzdani and Govindan [35] introduced a non-linear mathematical model to design a sustainable perishable FSC. In this study, the perishability of the products followed the Weibull distribution and was affected by the use of vehicle refrigerator. Goodarzian et al. [36] formulated a bi-objective mixed-integer non-linear programming model to design a citrus fruit supply chain network. The contribution of this paper is the development of a mathematical model for designing a three-echelon network that takes into account coefficient water, CO2 emissions, and time window concurrently. Kalantari and Hosseininezhad [12] developed a sustainable model considering four risk factors: delays, fluctuation of exchange rates, quality of raw materials and production. The authors used the cross-entropy method to solve the model for large instances using the optimization software GAMS. Momenitabar et al. [37] introduced a sustainable closed-loop supply chain network that minimizes total and environmental costs, including energy consumption and pollution emissions, and maximizes employment opportunities. To reduce the environmental impacts, the circular economy concept has been addressed in this study. Momenitabar et al. [38] developed a mathematical model for designing a sustainable bioethanol supply chain network. In order to determine the best method to predict the actual amount of demand, they compared three popular machine-learning methods. In addition, the authors considered two meta-heuristic algorithms to find pareto optimum solutions for the large-scale problem. Goodarzian et al. [39] investigated a novel multi-objective mixed-integer linear programming model to establish a sustainable citrus closed-loop supply chain network. The authors included the circularity strategy and the triple bottom lines of sustainability into their model. Mohammadi et al. [40] presented a mixed-integer non-linear programming model to design a sustainable FSC for perishable products. To show the validity of proposed model, they conducted several sensitivity analyses on crucial parameters and applied the model in a food company. Lastly, Momenitabar et al. [41] integrated machine-learning algorithms with quantitative optimization modeling to design a sustainable bioethanol supply chain. In this work, three machine-learning approaches have been applied to forecast the bioethanol demand as model inputs.

### 2.3. Risk-averse SCND

A few studies have considered risk-aversion in SCND and their mathematical models, some of which are presented in this subsection. Noyan [42] used two-stage stochastic programming (TSSP) to design a disaster management SC under disruption and operational risks. In order to address the variability of non-deterministic parameters, the author incorporated the CVaR measure into the stochastic programming framework. Ahmadi-Javid and Seddighi [43] developed a location-routing optimization model under disruption risks to design a SC consisting of manufacturers and distributors in which a single type of commodity is distributed to several consumers. The problem was formulated as a mixed-integer linear programming model under three risk measurement policies: optimistic, cautious, and pessimistic. The authors utilized the expectation, CVaR, and worst-case risk measures to calculate the risk under each policy. Soleimani and Govindan [44] used a risk-averse TSSP method to plan and design a reverse SC in which the CVaR was used to evaluate the risk associated with the uncertainty in prices and demand for returned products. Govindan and Fattahi [45] proposed a multi-period optimization model under demand uncertainty with the goal of minimizing the total SC cost. The problem was formulated by incorporating TSSP and the CVaR into the model to address risk-aversion. Rahimi and Ghezavati [46] developed a mixed-integer linear programming model to design and plan a sustainable reverse logistics network under uncertainty. The authors applied a risk-averse TSSP approach, utilized the CVaR to cope with the deviation of demand and investment rate from their expected values, and solved a numerical example to obtain potential managerial insights. Rahimi et al. [47] investigated risk-averse SCND by proposing a mixed-integer non-linear programming model to configure a sustainable SC in which the CVaR risk measure was assimilated into the model. This paper contributes mainly by offering various discount policies for selling products to customers. Lotfi et al. [48] developed a model to design an antifragile, sustainable, and agile network that integrates these concepts taking into account several factors such as resilience, risk, robustness, and environmental issues. The authors incorporated the Entropic Value at Risk (EVaR) measure into the stochastic programming framework to address risk-aversion. The cost function of this model encompasses the expected value, maximum, and EVaR. Lotfi et al. [49] proposed a novel hybrid method of robust optimization which employs a stochastic approach to address uncertainty and risk management. The objective function of the model given involves maximizing the coefficients of mean and minimum profits under multiple scenarios, while considering an energy-aware constraint. Lotfi et al. [50] introduced a novel viable SC in which robust stochastic optimization is combined with CVaR as risk criteria to ensure an optimal approach. The objective function of their model minimizes the expected value, maximum, and CVaR of cost. Lotfi et al. [51] presented a new data-driven robust optimization in viable supply chain network design by utilizing Open Innovation (OI) and Blockchain Technology (BCT). The authors employed a robust stochastic optimization technique and assimilated the CVaR measure into the model to effectively tackle uncertainty as well as risks.

### 2.4. Research gap

**Fig 1:**
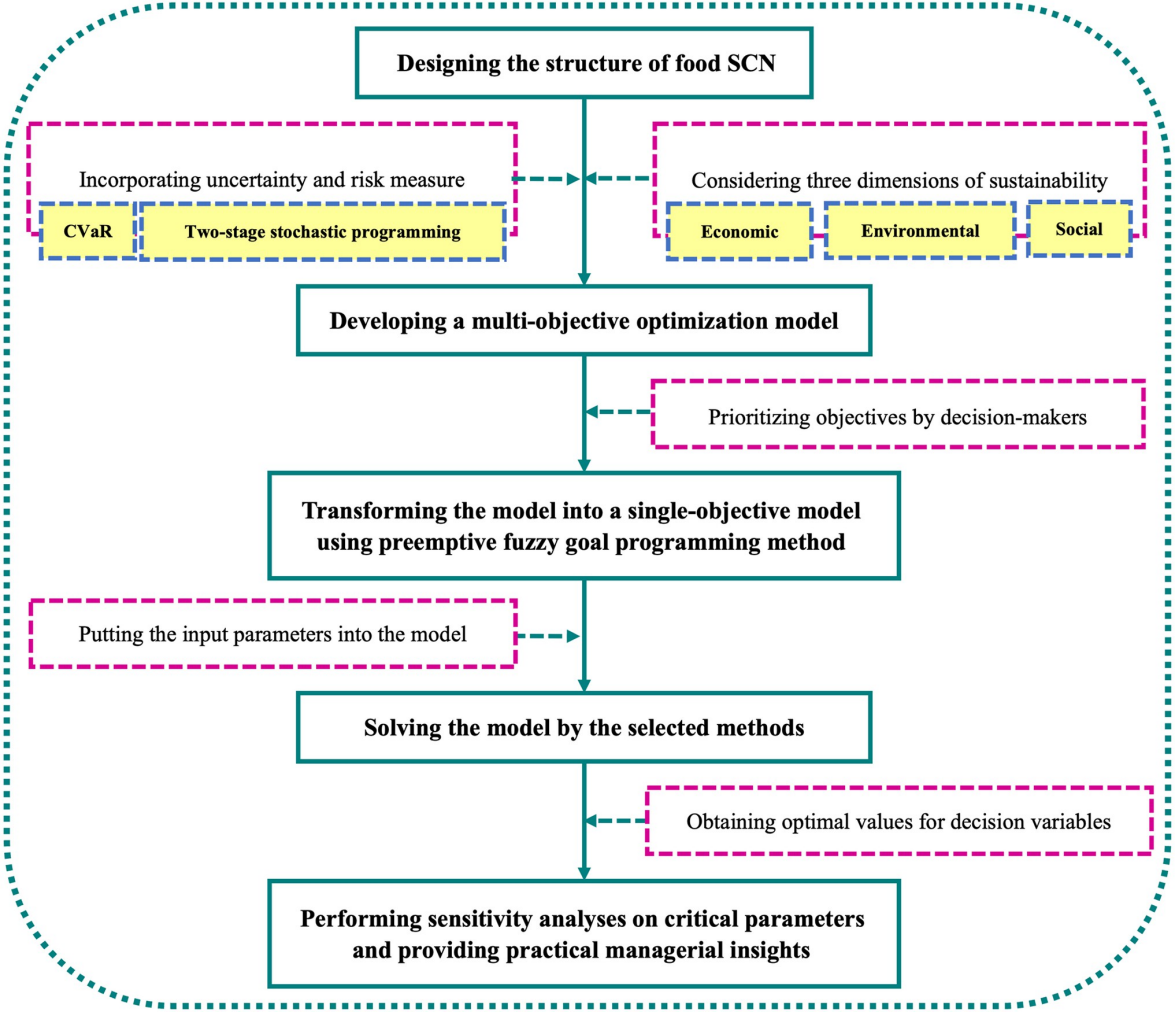
Methodology of the present study.

As our review of the literature reveals, several studies have investigated sustainable FSCND, but no comprehensive study has been conducted on risk-averse SCND while developing a sustainable FSC at the same time. To address this gap, we present a sustainable risk-averse FSC by incorporating the CVaR into our model to develop a robust network in an uncertain environment. Moreover, a few papers have considered the specific characteristics of food products, such as perishability, in their proposed FSCs (e.g. [13, 30, 34]). Therefore, we also incorporate perishability and its implications into our model by considering the shelf-life of foodstuff to prevent the decay of finished products along the SC. We discovered that making inventory and transportation decisions based on integration of production and delivery times may bridge a significant gap in the literature on FSCND. Furthermore, in the majority of the research papers on sustainability, the only instance of social impact addressed by the authors appeared to be job creation, and other factors such as safety had been largely neglected. Our research methodology outlining the steps undertaken is summarized by the flowchart presented as Fig 1, acting as a guide for the research process. In light of the gap analysis presented in this subsection, and based on Table 1, the present study may contribute to the literature in the following ways:

- Utilizing the CVaR measure for risk management to develop a robust, sustainable, risk-averse FSC;
- Considering perishability in inventory decisions based on the production times, delivery times, and shelf-life of the products;
- Investigating product transportation decisions in terms of both economic and environmental implications in FSC
- Considering job creation and safety together as the social impacts in the FSC;
- Proposing PFGP as a novel extension of goal programming to solve the presented multi-objective model;
- Verifying the applicability and validity of the proposed model and solution approach through a case study from the processed food industry.

## 3. Problem statement

**Fig 2:**
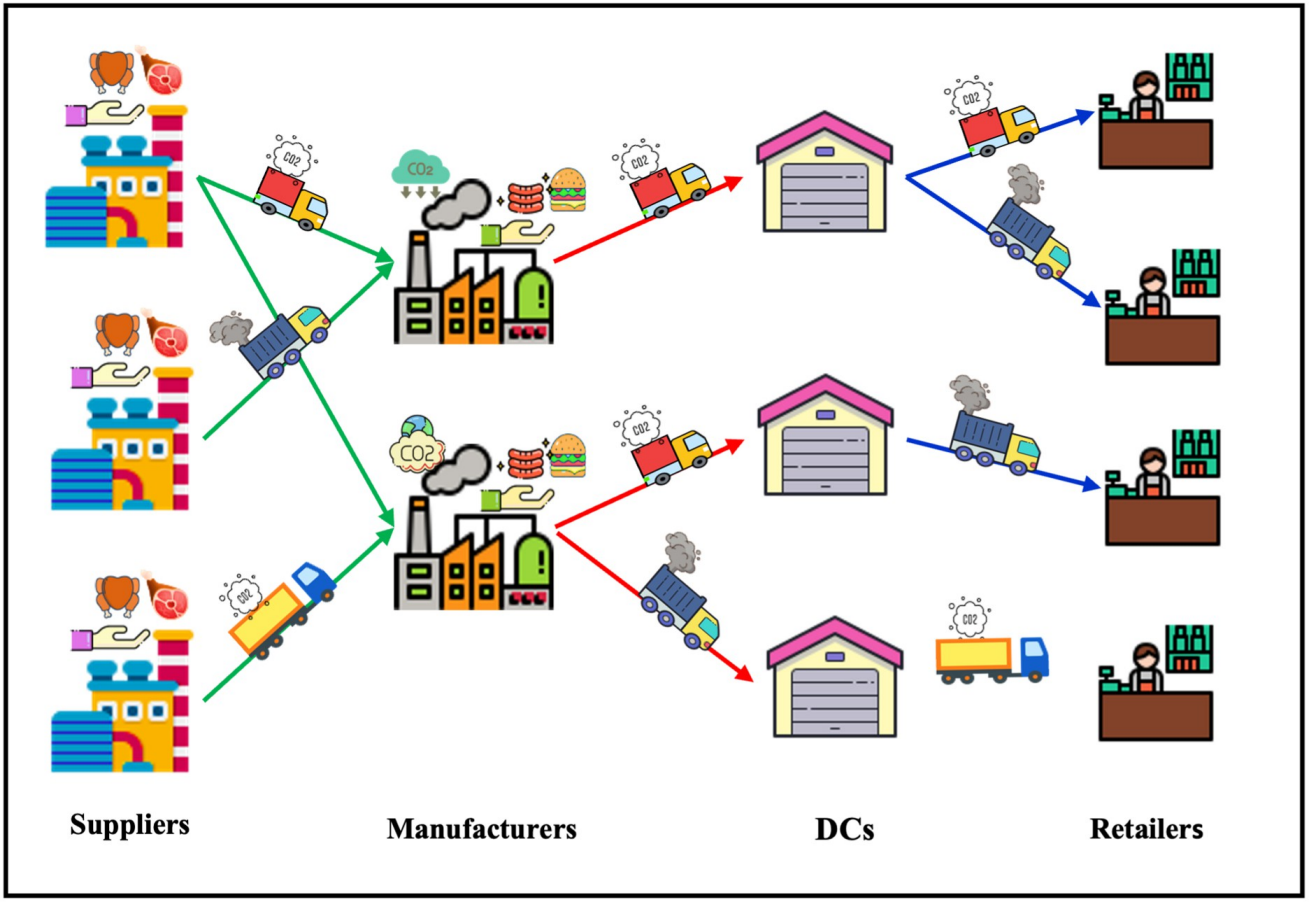
Structure of the proposed FSC. Republished from icons8.com under a CC BY license, with permission from Icons8, original copyright 2024.

In this study, we develop a risk-averse sustainable FSC considering the perishability of the food products. The proposed SC is multi-echelon, multi-period, and multi-product, and consists of raw material suppliers, manufacturers, distribution centers (DCs), and retailers with uncertain demand. The process of material flow begins by purchasing raw materials from the suppliers. Next, the products are manufactured in the factories and shipped to the retailers through the DCs by transportation modes. Fig 2 graphically illustrates the structure of the proposed FSC. To design the aforementioned network, a two-stage multi-objective programming model is presented. In this model, the three dimensions of sustainability are introduced in the form of three objective functions. In the first objective, the economic dimension of sustainability is addressed by minimizing the total costs of the network composed of the first stage costs, second stage costs, and CVaR costs. The environmental dimension of sustainability is the second objective function which minimizes the total environmental effects of the network. Total environmental effects of the SCN are comprised of carbon released by establishing facilities, production, handling processes and transportation. Lastly, social dimension of sustainability is considered in the third objective which represents the social aspect of the SC and aims at maximizing the total social effects of the network, which includes of created job opportunities and worker’s lost days multiplied to their given weight.

### 3.1. Assumptions

We formulated the model based on the following basic assumptions:

1. The potential locations to establish manufacturers and DCs are predetermined.
2. The capacity of suppliers, manufacturers, and DCs is limited.
3. Retailers can fulfill their demand only through DCs.
4. The demand of retailers is considered as a non-deterministic parameter.
5. Shortage is allowed in the model.
6. The transportation modes are heterogeneous with different capacities and carbon footprints.
7. The products are perishable and have a fixed shelf-life.
8. The decision-makers are only concerned with the costs in the second stage of stochastic programming.

### 3.2. Mathematical formulation

In this section we introduced the mathematical formulation of the described problem which is as follows (Table 2):

**Table 2:** Notation list.

**Sets (indices)**	
*I*	Set of suppliers indexed by *i*, (*i* ∈ *I*)
*J*	Set of potential locations for manufacturers indexed by *j*, (*j* ∈ *J*)
*K*	Set of potential locations for DCs indexed by *k*, (*k* ∈ *K*)
*H*	Set of retailers indexed by *h*, (*h* ∈ *H*)
*RA*	Set of raw materials indexed by *ra*, (*ra* ∈ *RA*)
*P*	Set of products indexed by *p*, (*p* ∈ *P*)
*M*	Set of transportation modes indexed by *m*, (*m* ∈ *M*)
*N*	Set of capacity levels for manufacturers indexed by *n*, (*n* ∈ *N*)
*L*	Set of capacity levels for DCs indexed by *l*, (*l* ∈ *L*)
*R*	Set of production periods indexed by *r*, (*r* ∈ *R*; *R* ⊂ *T*)
*E*	Set of delivery periods indexed by *e*, (*e* ∈ *E*; *E* ⊂ *T*)
*T*	Set of time periods indexed by *t*, (*t* ∈ *T*)
*S*	Set of scenarios indexed by *s*, (*s* ∈ *S*)
**Parameters**	
*fm*_*jn*_	Fixed cost of establishing manufacturer *j* with capacity level *n*
*fd*_*kl*_	Fixed cost of establishing DC *k* with capacity level *l*
*pc*_*ijrat*_	Per-unit purchasing cost of raw material *ra* from supplier *i* at time period *t*
*mc*_*pj*_	Per-unit production cost of product *p* by manufacturer *j*
*oc*_*pj*_	Per-unit operational cost of manufacturing product *p* by manufacturer *j*
*oc*_*pk*_	Per-unit operational cost of handling product *p* in DC *k*
*tc*_*ijm*_	Per-unit transportation cost from supplier *i* to manufacturer *j* using transportation mode *m*
*tc*_*jkm*_	Per-unit transportation cost from manufacturer *j* to DC *k* using transportation mode *m*
*tc*_*khm*_	Per-unit transportation cost from DC *k* to retailer *h* using transportation mode *m*
*h*_*jp*_	Per-unit holding cost of product *p* in manufacturer *j*
*h*_*kp*_	Per-unit holding cost of product *p* in DC *k*
*ls*_*pht*_	Per-unit cost of lost demand for product *p* in retailer *h* at time period *t*
* $d_{pht}^{s}$ *	Demand for product *p* in retailer *h* at time period *t* under scenario *s*
*sl*_*p*_	Shelf-life of product *p*
*cr*_*rap*_	Consumption rate of raw material *ra* per each unit of product *p*
*cap*_*ira*_	Maximum procurement capacity of supplier *i* for raw material *ra*
*cap*_*jpn*_	Maximum capacity of manufacturer *j* with capacity level *n* for product *p*
*cap*_*kpl*_	Maximum capacity of DC *k* with capacity level *l* for product *p*
*cap*_*m*_	Capacity of transportation mode *m*
*cp*_*pj*_	Production capacity of manufacturer *j* for product *p*
*ds*_*ij*_	Distance between supplier *i* and manufacturer *j* (kilometer)
*ds*_*jk*_	Distance between manufacturer *j* and DC *k* (kilometer)
*ds*_*kh*_	Distance between DC *k* and retailer *h* (kilometer)
*e*_*jn*_	Environmental impacts of establishing manufacturer *j* with capacity level *n*
*e*_*kl*_	Environmental impacts of establishing DC *k* with capacity level *l*
*ep*_*pjn*_	Environmental impacts of manufacturing product p in manufacturer *j* with capacity level *n*
*eh*_*pkl*_	Environmental impacts of handling product *p* in DC *k* with capacity level *l*
*et*_*m*_	Environmental impacts of transporting products or raw materials using transportation mode *m*
*jo*_*jn*_	Number of job opportunities created due to establishing manufacturer *j* with capacity level *n*
*jo*_*kl*_	Number of job opportunities created due to establishing DC *k* with capacity level *l*
*ld*_*jn*_	Number of days lost due to work-related damages while establishing manufacturer *j* with capacity level *n*
*ld*_*kl*_	Number of days lost due to work-related damages while establishing DC *k* with capacity level *l*
*wjo*	Weight given to job opportunities
*wld*	Weight given to workers’ lost days
*ω*_*s*_	Occurrence probability of scenario *s*
*θ*	Weight given to CVaR
*β*	CVaR confidence level
**Decision variables**	
*y*_*jn*_	A binary variable that equals 1 if manufacturer *j* with capacity level *n* is established; otherwise 0
*y*_*kl*_	A binary variable that equals 1 if DC *k* with capacity level *l* is established; otherwise 0
*y*_*m*_	A binary variable that equals 1 if transportation mode *m* is selected; otherwise 0
* $pr_{jpt}^{s}$ *	Quantity of product *p* produced by manufacturer *j* at time period *t* under scenario *s*
* $x_{\;ijramt}^{s}$ *	Quantity of raw material *ra* shipped from supplier *i* to manufacturer *j* using transportation mode *m* at time period *t* under scenario *s*
* $x_{jkpmt}^{s}$ *	Quantity of product *p* shipped from manufacturer *j* to DC *k* using transportation mode *m* at time period *t* under scenario *s*
* $x_{khpmt}^{s}$ *	Quantity of product *p* shipped from DC *k* to retailer *h* using transportation mode *m* at time period *t* under scenario *s*
* $u_{jkprt}^{s}$ *	Quantity of product *p* shipped from manufacturer *j* to DC *k* at time period *t* which is produced in period *r* under scenario *s*
* $u_{khpret}^{s}$ *	Quantity of product *p* shipped from DC *k* to retailer *h* at time period *t* which is produced at period *r* and delivered at period *e* under scenario *s*
* $in_{jptr}^{s}$ *	Inventory level of product *p* in manufacturer *j* until end of time period *t* which is produced in period *r* under scenario *s*
* $in_{kptre}^{s}$ *	Inventory level of product *p* in DC *k* until end of time period *t* which is produced in period *r* and delivered in period *e* under scenario *s*
* $xsh_{hpt}^{s}$ *	Quantity of lost demand for product *p* in retailer *h* at time period *t* under scenario *s*
*ϑ*_*s*_	Auxiliary variable for calculating CVaR
*η*	Free variable for calculating Value-at-risk (VaR)

**Mathematical model**:

**Objective functions**

$$\text{Min}\;{\text{f}}_{1}:\text{Total}\;\text{cost}=(1+\theta )\times \text{first}\;\text{stage}\;\text{costs}+\text{second-stage}\;\text{costs}+\theta \times \text{CVaR}\;\text{cost}$$ (1)

- **Economic objective function**

We apply a TSSP approach to formulate the problem. In this type of framework, decisions are made over two sequential stages. In the first stage, decision-makers should make scenario-independent decisions that typically determine the number, location, and capacity of facilities. Scenario-independent decisions are made before the realization of uncertain parameters (e.g. demand, supply, transportation costs, etc.) and generate the first-stage (a.k.a. design stage) costs. In the second stage, scenario-dependent decisions (e.g. material flow, inventory level, production rate, etc.) are made regarding the second stage (a.k.a. planning stage) costs after the values of uncertain parameters have been realized [52].

Eq (1) gives the first objective function, i.e. economic sustainability, which aims to minimize total cost of the SC, consisting of first-stage, second-stage, and CVaR costs. The components of Eq (1) are formulated as follows:

$$\text{First}\;\text{stage}\;\text{costs}=\sum_{j}\;\sum_{n}\;fm_{jn}y_{jn}+\sum_{k}\;\sum_{l}\;fd_{kl}y_{kl}$$ (2)

$$\begin{array}{ll} \text{Second}\;\text{stage}\;\text{costs} & =\sum_{i}\;\sum_{j}\;\sum_{ra}\;\sum_{m}\;\sum_{t}\;\sum_{s}\;pc_{ijrat}x_{ijramt}^{s}\omega_{s}\\ & +\sum_{j}\;\sum_{p}\;\sum_{t}\;\sum_{s}\;mc_{pj}pr_{jpt}^{s}\omega_{s}\\ & +\sum_{j}\;\sum_{k}\;\sum_{p}\;\sum_{m}\;\sum_{t}\;\sum_{s}\;oc_{pj}x_{jkpmt}^{s}\omega_{s}\\ & +\sum_{k}\;\sum_{h}\;\sum_{p}\;\sum_{m}\;\sum_{t}\;\sum_{s}\;oc_{pk}x_{khpmt}^{s}\omega_{s}\\ & +\sum_{j}\;\sum_{p}\;\sum_{r}\;\sum_{t|r\leq t}\;\sum_{s}\;h_{jp}in_{jptr}^{s}\omega_{s}\\ & +\sum_{k}\;\sum_{p}\;\sum_{r}\;\sum_{e|r\leq e}\;\sum_{t|r\leq t.e\leq t}\;\sum_{s}\;h_{kp}in_{kptre}^{s}\omega_{s}\\ & +\sum_{i}\;\sum_{j}\;\sum_{ra}\;\sum_{m}\;\sum_{t}\;\sum_{s}\;tc_{ijm}ds_{ij}x_{ijramt}^{s}\omega_{s}\\ & +\sum_{j}\;\sum_{k}\;\sum_{p}\;\sum_{m}\;\sum_{t}\;\sum_{s}\;tc_{jkm}ds_{jk}x_{jkpmt}^{s}\omega_{s}\\ & +\sum_{k}\;\sum_{h}\;\sum_{p}\;\sum_{m}\;\sum_{t}\;\sum_{s}\;tc_{khm}ds_{kh}x_{khpmt}^{s}\omega_{s}\\ & +\sum_{h}\;\sum_{p}\;\sum_{t}\;\sum_{s}\;ls_{pht}xsh_{hpt}^{s}\omega_{s} \end{array}$$ (3)

$$\text{CVaR}\;\text{costs}=\eta +\frac{1}{1-\beta }\sum_{s}\;\vartheta_{s}\omega_{s}$$ (4)

Eq (2) obtains the fixed costs of establishing manufacturers and DCs. Eq (3) calculates the second-stage costs, consisting of the costs of purchasing raw materials, production, distribution, inventory holding in manufacturers and DCs, transportation, and lost demand in retailers, with respect to the set of scenarios. The last component of Eq (1) relates to the costs of the CVaR, which is defined as Eq (4) [22].

- **Environmental objective function**

$$\begin{array}{ll} \text{Min}\;{\text{f}}_{2}:\text{Total}\;\text{environmental}\;\text{impacts} & =\text{carbon}\;\text{released}\;\text{by}\;\text{establishing}\;\text{facilities}\\ & +\text{carbon}\;\text{released}\;\text{by}\;\text{production}\\ & +\text{carbon}\;\text{released}\;\text{by}\;\text{production}\\ & +\text{carbon}\;\text{released}\;\text{by}\;\text{transportation} \end{array}$$ (5)

Eq (5) states the second objective which represents environmental sustainability and minimizes the total environmental impacts of the proposed SC, comprising the carbon emitted in the processes of establishing the facilities, production, handling, and transportation of the products. The terms of Eq (5) are presented as follows:

$$\text{Carbon}\;\text{emitted}\;\text{by}\;\text{establishing}\;\text{facilities}=\sum_{j}\;\sum_{n}\;e_{jn}y_{jn}+\sum_{k}\;\sum_{l}\;e_{kl}y_{kl}$$ (6)

$$\text{Carbon}\;\text{emitted}\;\text{by}\;\text{production}=\sum_{j}\;\sum_{p}\;\sum_{n}\;\sum_{t}\;\sum_{s}\;ep_{pjn}pr_{jpt}^{s}\omega_{s}$$ (7)

$$\text{Carbon}\;\text{emitted}\;\text{by}\;\text{handling}\;\text{processes}=\sum_{j}\;\sum_{k}\;\sum_{p}\;\sum_{l}\;\sum_{m}\;\sum_{t}\;\sum_{s}\;eh_{pkl}x_{jkpmt}^{s}\omega_{s}$$ (8)

$$\begin{array}{ll} \text{Carbon}\;\text{emitted}\;\text{by}\;\text{transportation} & =\sum_{i}\;\sum_{j}\;\sum_{ra}\;\sum_{m}\;\sum_{t}\;\sum_{s}\;et_{m}ds_{ij}x_{ijramt}^{s}\omega_{s}\\ & +\;\sum_{j}\;\sum_{k}\;\sum_{p}\;\sum_{m}\;\sum_{t}\;\sum_{s}\;et_{m}ds_{jk}x_{jkpmt}^{s}\omega_{s}\\ & +\sum_{j}\;\sum_{k}\;\sum_{p}\;\sum_{m}\;\sum_{t}\;\sum_{s}\;et_{m}ds_{kh}x_{khpmt}^{s}\omega_{s} \end{array}$$ (9)

Eq (6) computes the amount of carbon emitted in the process of establishing the manufacturers and DCs. Eqs (7) and (8) compute the environmental impacts of production and handling processes in the manufacturers and DCs, respectively. Eq (9) determines the amount of carbon emitted from the transportation of products. The first term of Eq (9) states the environmental impacts of transporting raw materials from suppliers to manufacturers, while the second term represents the environmental impacts of transporting the finished products from manufacturers to DCs. The last term of Eq (9) calculates the environmental impacts of transporting the products from DCs to retailers.

- **Social objective function**

$$\begin{array}{ll} \text{Max}\;{\text{f}}_{3}:\text{Total}\;\text{social}\;\text{impacts} & =wjo\times \text{number}\;\text{of}\;\text{created}\;\text{job}\;\text{opportunities}\\ & -wld\times \text{number}\;\text{of}\;\text{worker’s}\;\text{lost}\;\text{days} \end{array}$$ (10)

Eq (10) concerns social sustainability and aims to maximize the total social impacts of the proposed SC which consists of the number of job opportunities created and workers’ lost days multiplied by their weight. The components of Eq (10) are defined as follows:

$$\text{Number}\;\text{of}\;\text{jobs}\;\text{created}=\sum_{j}\;\sum_{n}\;jo_{jn}y_{jn}+\sum_{k}\;\sum_{l}\;jo_{kl}y_{kl}$$ (11)

$$\text{Number}\;\text{of}\;\text{workers'}\;\text{lost}\;\text{days}=\sum_{j}\;\sum_{n}\;ld_{jn}y_{jn}+\sum_{k}\;\sum_{l}\;ld_{kl}y_{kl}$$ (12)

The first component of Eq (10) states the number of jobs created by establishing manufacturers and DCs, presented as Eq (11). The second component, calculated by Eq (12), pertains to the number of lost days caused by work-related damages during the process of establishing manufacturers and DCs.

**Constraints**

$$\sum_{n}\;y_{jn}\leq 1\;\forall \;j$$ (13)

$$\sum_{l}\;y_{kl}\leq 1\;\forall \;k$$ (14)

$$\sum_{i}\;\sum_{m}\;x_{ijramt}^{s}\leq \sum_{p}\;cr_{rap}pr_{jpt}^{s}\;\forall \;j,ra,t,s$$ (15)

$$\sum_{r=1}^{t}\;in_{jptr}^{s}=pr_{jpt}^{s}-\sum_{k}\;\sum_{m}\;x_{jkpmt}^{s}\;\forall \;j,p,t=1<sl_{p},s$$ (16)

$$\sum_{r=1}^{t}\;in_{jptr}^{s}=\sum_{r=1}^{t-1}\;in_{jp\left(t-1\right)r}^{s}+pr_{jpt}^{s}-\sum_{k}\;\sum_{m}\;x_{jkpmt}^{s}\;\forall \;j,p,1<t<sl_{p},s$$ (17)

$$\sum_{r=t+1-sl_{p}}^{t}\;in_{jptr}^{s}=\sum_{r=t+1-sl_{p}}^{t-1}\;in_{jp\left(t-1\right)r}^{s}+pr_{jpt}^{s}-\sum_{k}\;\sum_{m}\;x_{jkpmt}^{s}\;\forall \;j,p,t\geq sl_{p},s$$ (18)

$$\sum_{m}\;x_{jkpmt}^{s}=\sum_{r=1}^{t}\;u_{jkprt}^{s}\;\forall \;j,k,p,t<sl_{p},s$$ (19)

$$\sum_{m}\;x_{jkpmt}^{s}=\sum_{r=t+1-sl_{p}}^{t}\;u_{jkprt}^{s}\;\forall j,k,p,t\geq sl_{p},s$$ (20)

$$in_{jptr}^{s}=pr_{jpt}^{s}-\sum_{k}\;u_{jkprt}^{s}\;\forall j,p,r,t,s,r=t$$ (21)

$$in_{jptr}^{s}=in_{jp\left(t-1\right)r}^{s}-\sum_{k}\;u_{jkprt}^{s}\;\forall j,p,r,t,s,t-r<sl_{p}$$ (22)

$$\sum_{e=r}^{t}\;\sum_{r=1}^{t}\;in_{kptre}^{s}=\sum_{j}\;\sum_{m}\;x_{jkpmt}^{s}-\sum_{h}\;\sum_{m}\;x_{khpmt}^{s}\;\forall k,p,t=1<sl_{p},s$$ (23)

$$\begin{array}{cc} \begin{array}{c} \sum_{e=r}^{t}\;\sum_{r=1}^{t}\;in_{kptre}^{s}=\sum_{e=r}^{t-1}\;\sum_{r=1}^{t-1}\;in_{kp\left(t-1\right)re}^{s}+\sum_{j}\;\sum_{m}\;x_{jkpmt}^{s}\\ -\sum_{h}\;\sum_{m}\;x_{khpmt}^{s} \end{array} & \forall k,p,1<t<sl_{p},s \end{array}$$ (24)

$$\begin{array}{cc} \begin{array}{c} \sum_{e=r}^{r+sl_{p}-1}\;\sum_{r=t-sl_{p}+1}^{t}\;in_{kptre}^{s}=\sum_{e=r}^{t-1}\;\sum_{r=t-sl_{p}+1}^{t-1}\;in_{kp\left(t-1\right)re}^{s}\\ +\sum_{j}\;\sum_{m}\;x_{jkpmt}^{s}-\sum_{h}\;\sum_{m}\;x_{khpmt}^{s} \end{array} & \forall k,p,t\geq sl_{p},s \end{array}$$ (25)

$$\sum_{m}\;x_{khpmt}^{s}=\sum_{e=r}^{t}\;\sum_{r=1}^{t}\;u_{khpret}^{s}\;\forall k,h,p,t<sl_{p},s$$ (26)

$$\sum_{m}\;x_{khpmt}^{s}=\sum_{e=r}^{r+sl_{p}-1}\;\sum_{r=t-sl_{p}+1}^{t}\;u_{khpret}^{s}\;\forall k,h,p,t\geq sl_{p},s$$ (27)

$$in_{kptre}^{s}=\sum_{j}\;u_{jkprt}^{s}-\sum_{h}\;u_{khpret}^{s}\;\forall k,p,r,e,t,s,e=t$$ (28)

$$in_{kptre}^{s}=in_{kp\left(t-1\right)re}^{s}-\sum_{h}\;u_{khpret}^{s}\;\forall k,p,r,e,t,s,t-e<sl_{p}$$ (29)

$$\sum_{k}\;\sum_{m}\;x_{khpmt}^{s}+xsh_{hpt}^{s}\geq d_{pht}^{s}\;\forall h,p,t,s$$ (30)

$$\sum_{j}\;\sum_{m}\;x_{ijramt}^{s}\leq cap_{ira}\;\forall i,ra,t,s$$ (31)

$$pr_{jpt}^{s}\leq cp_{pj}\;\forall j,p,t,s$$ (32)

$$\sum_{k}\;\sum_{m}\;x_{jkpmt}^{s}\leq \sum_{n}\;cap_{jpn}y_{jn}\;\forall j,p,t,s$$ (33)

$$\sum_{h}\;\sum_{m}\;x_{khpmt}^{s}\leq \sum_{l}\;cap_{kpl}y_{kl}\;\forall k,p,t,s$$ (34)

$$\sum_{ra}\;x_{ijramt}^{s}\leq cap_{m}y_{m}\;\forall i,j,m,t,s$$ (35)

$$\sum_{p}\;x_{jkpmt}^{s}\leq cap_{m}y_{m}\;\forall j,k,m,t,s$$ (36)

$$\sum_{p}\;x_{khpmt}^{s}\leq cap_{m}y_{m}\;\forall k,h,m,t,s$$ (37)

$$in_{jptr}^{s}=0\;\forall j,p,t,r,s,t<r$$ (38)

$$in_{kptre}^{s}=0\;\forall k,p,t,r,e,s,t<r$$ (39)

$$in_{kptre}^{s}=0\;\forall k,p,t,r,e,s,e<r$$ (40)

$$\begin{array}{lll} \vartheta_{s} & \geq \sum_{i}\;\sum_{j}\;\sum_{ra}\;\sum_{m}\;\sum_{t}\;pc_{ijrat}x_{ijramt}^{s}+\sum_{j}\;\sum_{p}\;\sum_{t}\;mc_{pj}pr_{jpt}^{s} & \forall s\\ & +\sum_{j}\;\sum_{p}\;\sum_{r}\;\sum_{t|r\leq t}\;h_{jp}in_{jptr}^{s}+\sum_{k}\;\sum_{p}\;\sum_{r}\;\sum_{e|r\leq e}\;\sum_{t|r\leq t.e\leq t}\;h_{kp}in_{kptre} & \\ & +\sum_{j}\;\sum_{k}\;\sum_{p}\;\sum_{m}\;\sum_{t}\;oc_{pj}x_{jkpmt}^{s}+\sum_{k}\;\sum_{h}\;\sum_{p}\;\sum_{m}\;\sum_{t}\;oc_{pk}x_{khpmt}^{s} & \\ & +\sum_{i}\;\sum_{j}\;\sum_{ra}\;\sum_{m}\;\sum_{t}\;tc_{ijm}ds_{ij}x_{ijramt}^{s}+\sum_{j}\;\sum_{k}\;\sum_{p}\;\sum_{m}\;\sum_{t}\;tc_{jkm}ds_{jk}x_{jkpmt}^{s} & \\ & +\sum_{k}\;\sum_{h}\;\sum_{p}\;\sum_{m}\;\sum_{t}\;tc_{khm}ds_{kh}x_{khpmt}^{s}+\sum_{h}\;\sum_{p}\;\sum_{t}\;ls_{pht}xsh_{hpt}^{s}-\eta & \end{array}$$ (41)

$$y_{jn},y_{kl},y_{m}\in \;\left\{0,1\right\}\;\forall j,n,k,l,m$$ (42)

$$\begin{array}{ll} \begin{array}{l} pr_{jpt}^{s},x_{ijramt}^{s},x_{jkpmt}^{s},x_{khpmt}^{s},u_{jkprt}^{s},u_{khpret}^{s},\\ \;in_{jptr}^{s},in_{kptre}^{s},xsh_{hpt}^{s},\vartheta_{s}\geq 0 \end{array} & \forall i,j,k,h,ra,p,m,r,e,t,s \end{array}$$ (43)

Eqs (13) and (14) ensure that each manufacturer and DC can be established only at one capacity level. Eq (15) indicates the amount of raw material purchased from suppliers. Due to the fact that food products are inherently perishable, inventory management and transportation decisions between network nodes require applying more constraints compared to other products. Therefore, in this paper, by considering the shelf life, production and delivery times of products individually, we intend to prevent the perishability of products along the network. In fact, the level of products inventory and the amount of products flow between network nodes are associated with the optimal production and delivery times as well as shelf life of the products. To address this issue, Eqs (16) to (29) have been defined in the proposed model. Eqs (16) to (18) calculate the inventory level of products according to their shelf-life for each manufacturer at different periods. Eqs (16) and (17) show the level of on-hand inventory at time periods shorter than the shelf-life of the product. However it is worth mentioning that in the first period, no inventory is added from the previous period. Therefore, the inventory levels of the products in each manufacturer for *t* = 1 < *sl*_*p*_ and 1 < *t* < *sl*_*p*_ are calculated separately using Eqs (16) and (17), respectively. Eq (18) computes the inventory level at time periods longer than the shelf-life of the product. Eqs (19) and (20) compute the flow of each product from a manufacturer to a DC considering its production period. As mentioned in Eqs (16) to (18), the product flows between the relevant nodes are formulated differently for *t* < *sl*_*p*_ and *t* ≥ *sl*_*p*_. Eqs (21) and (22) present the inventory balance of products in each manufacturer based on their production period. Similar to Eqs (16) to (18), the inventory levels of products in the DCs for different intervals of *t* are calculated by Eqs (23) to (25). Eqs (26) and (27) define the flow of each product from a DC to a retailer based on its production time. Eqs (28) and (29) determine the inventory levels of DCs based on their delivery periods. Eq (28) indicates when the delivery period is equal to the time period, the inventory level of DCs is equal to the quantity of products delivered from manufacturers to DCs subtracted by the flow of products to retailers. Eq (29) states that when *t* − *e* < *sl*_*p*_, the current inventory level is equal to the inventory level of the previous period subtracted by the flow of products to the retailers. Eq (30) determines the quantity of products shipped to retailers as well as the quantity of shortage. Eq (31) defines the raw material procurement capacity of suppliers. Eq (32) presents the production capacity of each manufacturer at each time period. Eqs (34) and (35) express the maximum throughput capacity of each manufacturer and DC, respectively. Eqs (35) to (37) show the capacity constraint of the transportation modes. Eqs (38) to (40) state the decision variables whose values should be zero. Eq (41) calculates the deviational value that exceeds the value-at-risk. Finally, Eqs (42) and (43) express the types of the decision variables.

### 3.3. Solution approach

In the process of solving real-world problems, there is often more than one objective function, each offering a specific solution if optimized separately. Such circumstances require that a trade-off be established among the objective functions and multi-objective optimization approaches were originally conceived precisely to solve such problems. One of the most commonly-used optimization approaches, introduced by Charnes et al. [53], is known as goal programming. In this approach, decision-makers define an expectation level for each objective function as its goal and try to minimize the deviation from these expectation levels [54]. Ostensibly, decision-makers should accurately determine the expectation level of each objective; however, in reality, the uncertainty of the available information makes it nearly impossible to assign perfectly accurate levels to the objectives. Fuzzy sets theory, introduced by Zadeh [55], is regarded as an effective approach to decision-making under uncertainty. Arikan and Güngör [56] then combined fuzzy set theory and goal programming to create the fuzzy goal programming (FGP) approach. Recently, a novel extension of FGP, known as preemptive fuzzy goal programming (PFGP), was proposed by. The model was developed with different priorities and had the advantage of largely eliminating the limitations of the original approach. One such limitation was that decision-makers were required to set a precise lower bound for the satisfaction degree of the objectives. Moreover, it was often the case that in order to achieve the minimum satisfaction degree for one of the objectives, a better total satisfaction degree for all objectives had to be eliminated. The new model identified and mitigated this issue, accepting solutions that have a higher satisfaction degree while taking into account the priority of the objectives in question. In this approach, the goals are prioritized and the satisfaction degree of the first priority should be greater than the others. By adding certain equations to constraints that illustrate the importance of each goal, this priority structure is taken into account. PFGP is applicable to multi-objective problems that involve uncertainty and objectives with different importance degrees [57].

In the present study, we adopt the PFGP method as it eliminates the requirement that a fixed value be determined for both the objective function and the satisfaction degree. It also takes uncertainty into account when determining the goal of each objective. Using this method, decision-makers can easily adjust the priority of the objective functions and observe the results of the optimal solution [58]. The notations of PFGP are as follows (Table 3):

**Table 3:** Notations of PFGP model.

*λ*_*i*_	Satisfaction degree of objective function *i*
*f*_*i*_	Value of objective function *i*
*u*_*i*_	Upper bound of objective function *i*
*l*_*i*_	Lower bound of objective function *i*
*g*_*i*_	Goal of objective function *i*
$s_{i}^{+}$	Positive deviation from the expected level
$s_{i}^{-}$	Negative deviation from the expected level

Using the notations presented above, the mathematical model based on PFGP can be expressed as follows:

$$Max\;Z=\sum_{i}\;\lambda_{i}$$ (44)

*s*.*t*.


$$h_{c}\left(X\right)=\left(\leq \;\;\;\geq \right)0\;\forall c$$ (45)

$$\lambda_{i}+\frac{1}{u_{i}-g_{i}}\times s_{i}^{+}\leq 1\;\forall i=\left(1,2,\ldots ,ii\right)$$ (46)

$$f_{i}-s_{i}^{+}\leq g_{i}\;\forall i=\left(1,2,\ldots ,ii\right)$$ (47)

$$\lambda_{i}+\frac{1}{g_{i}-l_{i}}\times s_{i}^{-}\leq 1\;\forall i=\left(ii+1,\ldots ,i\right)$$ (48)

$$f_{i}+s_{i}^{-}\geq g_{i}\;\forall i=\left(ii+1,\ldots ,i\right)$$ (49)

$$\lambda_{i}\geq \lambda_{í}\;\forall i\neq í$$ (50)

$$X,\;\lambda_{i},\;s_{i}^{+},\;s_{i}^{-}\geq 0\;$$ (51)

Eq (44) expresses the goal of the PFGP approach which is maximizing the total satisfaction degree. In Eq (45), *h*_*c*_(*X*) represents the constraints of the main model. Eqs (46) and (47) determine the satisfaction degrees of the goals for minimization objectives, where ∀*i* = (1, 2,…, *ii*) is the set of minimization objectives. Eqs (48) and (49) calculate the satisfaction degrees of the goals for maximization objectives, where ∀*i* = (*ii* + 1,…, *i*) is the set of maximization objectives. Eq (50) relates to the priority of the goals, stating that the most important objective should achieve a satisfaction degree greater than or equal to the other objectives. Finally, Eq (51) defines the type of the decision variables of the PFGP model.

## 4. Results

In this section, we introduce the case study used in this research and the input data of its parameters. The problem is then solved using PFGP, and the analysis results are presented at the end.

### 4.1. Case description

Due to the constantly evolving lifestyles of consumers, the processed food industry plays a significant role in providing the population’s needs by producing and distributing a variety of products, also appearing to be on a growing trajectory. In this study, we investigate a processed food company as a real-life case study to measure the effectiveness of our model. The manufacturing plant is located in Tehran province, Iran, and the products are sold on domestic and international markets. The manufacturer procures its required raw materials, mainly chicken and beef, from multiple suppliers. In the proposed model, we consider only two types of finished products: hamburger 80% beef, and sausage 70% chicken. Because of their perishability, the products are transported by refrigerated trucks. The network size of the case study detailed in Table 4.

**Table 4:** Size of the SC.

Set	Index	Value
Suppliers	*i*	8
Potential manufacturers	*j*	3
Potential DCs	*k*	5
Retailers	*h*	12
Raw materials	*ra*	2
Products	*p*	2
Transportation modes	*m*	3
Capacity levels of manufacturers	*n*	3
Capacity levels of DCs	*l*	3
Production periods	*r*	6
Delivery periods	*e*	6
Time periods	*t*	6
Scenarios	*s*	3

In this study, we focus on three three modes of transportation: light truck, medium truck, and heavy truck. The data used in the present research on the capacities of transportation modes and the quantities of CO_2_ emissions have been retrieved from Mohammed et al. [25] and Nayeri et al. [59] (see Table 5). Three levels of capacity: low, medium, and high, with different costs, carbon emissions, and job opportunities have been considered for establishing facilities in potential locations. Tables 6 and 7 show the values of the parameters related to each manufacturer and DC with different capacity levels. Also, we examine three prominent demand scenarios for each retailer in our case study: the most pessimistic, the most probable, and the most optimistic. Numerous studies in the literature on SCND problems have repeatedly implemented the same three demand levels to generate their scenarios and have subsequently attested to the reliability and efficiency of the method (e.g. [22, 60, 61]). According to the historical data obtained from the case study as well as the experts interviewed for the purposes of this paper, the occurrence probability of scenarios are estimated at 0.25, 0.5, and 0.25, respectively. Tables 8 to 11 detail the costs of the model, including the fixed cost of establishing facilities, operational cost, raw material purchasing cost, and holding cost of finished products. Since the finished products discussed in this study have different raw material consumption rates, weights, shelf-lives, and production processes, the production capacity and costs associated with each type of product vary, as well (see Table 12). In addition, Table 13 provides the data on the corresponding values of each retailer’s demand under each scenario. The environmental parameters are presented in Tables 14 to 16. We have gathered the data on environmental parameters from several sources, including the food carbon footprint calculator, an online platform provided by MyEmissions.green, and also from academic articles (e.g. [62]). The values of the social parameters can be found in Tables 17 and 18.

**Table 5:** Data of the transportation modes operating in the SC.

*m*	(*cap*_*m*_) (kg)	(*et*_*m*_) (kg/km)	(*tc*_*ijm*_), (*tc*_*jkm*_), (*tc*_*khm*_) (× 10^4^ Rials/kg-km)
Light truck	5,000	0.048	1.25
Medium truck	10,000	0.252	0.58
Heavy truck	14,000	0.297	0.44

**Table 6:** Maximum capacity of manufacturers with different capacity levels (kg) for finished products.

(*cap*_*jpn*_)		*j*		
*n*	*p*	1	2	3
Low	Hamburger 80% beef	42,000	64,000	106,000
	Sausage 70% chicken	60,000	106,000	170,000
Medium	Hamburger 80% beef	50,000	75,000	125,000
	Sausage 70% chicken	70,000	125,000	200,000
High	Hamburger 80% beef	58,000	86,000	144,000
	Sausage 70% chicken	80,000	144,000	230,000

**Table 7:** Maximum capacity of DCs with different capacity levels (kg) for finished products.

(*cap*_*kpl*_)		*k*				
*l*	*p*	1	2	3	4	5
Low	Hamburger 80% beef	80,000	160,000	125,000	180,000	190,000
	Sausage 70% chicken	55,000	180,000	80,000	115,000	125,000
Medium	Hamburger 80% beef	90,000	180,000	140,000	200,000	210,000
	Sausage 70% chicken	60,000	120,000	90,000	130,000	140,000
High	Hamburger 80% beef	100,000	200,000	150,000	220,000	230,000
	Sausage 70% chicken	65,000	130,000	100,000	145,000	155,000

**Table 8:** Fixed cost of establishing manufacturers and DCs with different capacity levels (×10^9^ Rials).

(*fm*_*jn*_), (*fd*_*kl*_) *n*, *l*	*j*			*k*				
	1	2	3	1	2	3	4	5
Low	1,000	1,700	2,720	90	220	120	240	320
Medium	1,200	2,000	3,200	100	245	140	270	335
High	1,380	2,300	3,680	110	270	160	300	350

**Table 9:** Operational cost of manufacturers and DCs (×10^4^ Rials).

(*oc*_*pj*_), (*oc*_*pk*_) *p*	*j*			*k*				
	1	2	3	1	2	3	4	5
Hamburger 80% beef	15.2	15.2	15.2	3.8	3.8	3.8	3.8	3.8
Sausage 70% chicken	8	8	8	2	2	2	2	2

**Table 10:** Data of raw material suppliers.

*i*	*ra*	(*cap*_*ira*_) (kg)	(*pc*_*ijrat*_) (×10^4^ Rials)
1	Beef	280,000	135
	Chicken	−	−
2	Beef	432,000	131
	Chicken	−	−
3	Beef	410,000	140
	Chicken	−	−
4	Beef	205,000	136
	Chicken	−	−
5	Beef	−	−
	Chicken	9,500,000	49
6	Beef	−	−
	Chicken	1,500,000	45
7	Beef	−	−
	Chicken	1,000,000	47
8	Beef	−	−
	Chicken	900,000	46

**Table 11:** Holding cost of finished products in manufacturers and DCs (×10^4^ Rials).

(*h*_*jp*_), (*h*_*kp*_)	*j*			*k*				
*p*	1	2	3	1	2	3	4	5
Hamburger 80% beef	1.9	1.9	1.9	1.9	1.9	1.9	1.9	1.9
Sausage 70% chicken	1	1	1	1	1	1	1	1

**Table 12:** Production data of finished products by manufacturers.

*j*	*p*	(*cp*_*pj*_) (kg)	(*mc*_*pj*_) (×10^4^ Rials)
1	Hamburger 80% beef	145,000	12
	Sausage 70% chicken	130,000	17
2	Hamburger 80% beef	90,000	12
	Sausage 70% chicken	145,000	17
3	Hamburger 80% beef	160,000	12
	Sausage 70% chicken	180,000	17

**Table 13:** Demand for finished products (kg).

($d_{pht}^{s}$)						
*p*	Hamburger 80% beef			Sausage 70% chicken		
	*s*					
*h*	1	2	3	1	2	3
1	1,500	3,000	4,500	25,00	5,000	7,500
2	1,200	2,400	3,600	2,000	4,000	6,000
3	3,600	7,200	10,800	6,000	12,000	18,000
4	2,700	5,400	8,100	4,500	9,000	13,500
5	1,800	3,600	5,400	3,000	6,000	9,000
6	1,500	3,000	4,500	2,500	5,000	7,500
7	3,000	6,000	9,000	5,000	10,000	15,000
8	900	1,800	2,700	1,500	3,000	4,500
9	600	1,200	1,800	1,000	2,000	3,000
10	2,400	4,800	7,200	4,000	8,000	12,000
11	7,500	15,000	22,500	12,500	25,000	37,500
12	3,300	6,600	9,900	5,500	11,000	16,500

**Table 14:** Carbon emissions from establishing manufacturers and DCs with different capacity levels (kg).

(*e*_*jn*_), (*e*_*kl*_) *n*, *l*	*j*			*k*				
	1	2	3	1	2	3	4	5
Low	40,000	54,000	70,000	9,000	13,000	12,000	14,000	18,000
Medium	43,000	59,000	75,000	10,000	14,000	12,500	14,900	18,800
High	46,000	64,000	79,000	11,600	15,800	13,700	15,600	19,800

**Table 15:** Carbon emissions from production of finished products in manufacturers (kg).

(*ep*_*pjn*_) *p*	*j*		
	1	2	3
Hamburger 80% beef	0.43	0.43	0.43
Sausage 70% chicken	0.66	0.66	0.66

**Table 16:** Carbon emissions from handling finished products in DCs (kg).

(*eh*_*pkl*_) *p*	Hamburger 80% beef			Sausage 70% chicken		
	*l*					
*k*	Low	Medium	High	Low	Medium	High
1	0.28	0.32	0.35	0.25	0.28	0.33
2	0.35	0.37	0.40	0.31	0.40	0.41
3	0.32	0.35	0.37	0.35	0.37	0.40
4	0.37	0.39	0.42	0.34	0.35	0.38
5	0.39	0.41	0.44	0.35	0.38	0.41

**Table 17:** Number of job opportunities created in manufacturers and DCs with different capacity levels.

(*jo*_*jn*_), (*jo*_*kl*_) *n*, *l*	*j*			*k*				
	1	2	3	1	2	3	4	5
Low	250	320	440	40	60	50	70	80
Medium	310	400	520	45	70	55	80	95
High	360	470	585	50	80	60	90	110

**Table 18:** Number of lost days due to work-related damages in manufacturers and DCs with different capacity levels.

(*ld*_*jn*_), (*ld*_*kl*_) *n*, *l*	*j*			*k*				
	1	2	3	1	2	3	4	5
Low	6	4	5	2	2	3	3	4
Medium	8	7	7	4	3	5	4	5
High	10	9	11	5	6	7	5	6

### 4.2. Computational results

The proposed mathematical model is solved by the optimization software GAMS using the case study parameters. Furthermore, *λ*_1_ > *λ*_2_ > *λ*_3_ is the fuzzy satisfaction degree reflecting the importance degrees of the objective functions according to the experts’ opinions. Table 19 details the output generated from solving the model. In addition, the numerical results of some analyses performed on the model are presented in the following subsections.

**Table 19:** Model output details.

Satisfaction degree of economic objective function	0.891
Satisfaction degree of environmental objective function	0.765
Satisfaction degree of social objective function	0.486
Total satisfaction degrees	2.142
Value of economic objective function	1952306 × 10^7^
Value of environmental objective function	75179800
Value of social objective function	491.4

#### 4.2.1. Comparing model

**Fig 3:**
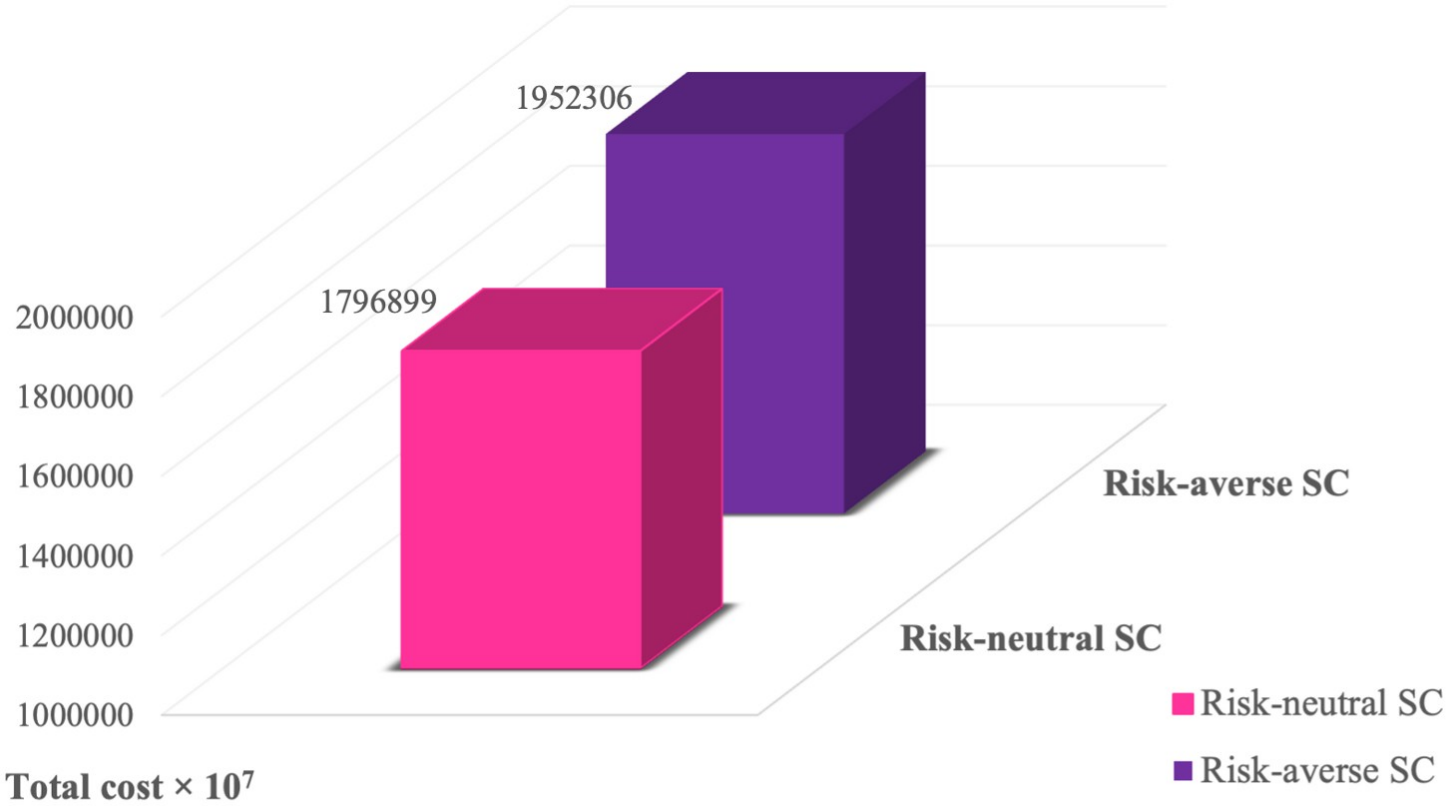
Comparison of risk-averse and risk-neutral problems.

The majority of stochastic programming models addressing food supply chain (FSC) under uncertainty are usually designed to be risk-neutral. In this paper, a risk-averse two-stage stochastic programming is aiming to trade off the expected total cost and risk cost, based on a predetermined level of responsive risk aversion employing CvaR as a coherent risk measure. The purpose of this subsection is to compare the main risk-averse problem (considering CvaR) and risk-neutral problem (without considering CvaR). The comparison of the economic objective function of the proposed model between risk-averse and risk-neutral problems is illustrated in Fig 3. As can be seen, the value of economic objective function in risk-averse problem is 8.65% more than risk-neutral one. Since it overcomes the variability of the uncertain parameters, provides more robust solutions compared to a risk-neutral problem in presence of uncertainty, and assist decision makers when faced decisions with uncertain outcomes. It is worth noting that decision-makers can achieve higher levels of risk aversion by increasing the value of network costs, which will cause the model to behave more conservatively and generate better solutions in worst-case scenarios.

#### 4.2.2. Analysis of lost demands

In this subsection, we intend to examine the service level of the SC in terms of lost demands. In the long run, unmet demands can result in customer dissatisfaction which, in turn, could negatively affect the firm’s reputation. Consequently, we can conclude that the more the level of service provided by the SC, the less lost demand will occur. In simple terms, lost demand is a consequence of increasing the total cost with the purpose of establishing balance between economic, social, and environmental sustainability. Table 20 details our analysis of the total value and percentage of lost demands for finished products under each scenario. The finished products in each scenario have varying amounts of unfulfilled demands in which the abovementioned reason is mainly responsible for them. Another noteworthy point inferred from the table is that when the demand for the finished products rises by 50% in the in probable scenario, the quantities of lost demand for the two products increase by 32.1% and 35.7%, respectively. It may thus be concluded that insufficient production capacity is the most likely culprit for the resulting unmet demand.

**Table 20:** Details of lost demands.

Scenario no.	Total demand for hamburger 80% beef	Total demand for sausage 70% chicken	Lost demand for hamburger 80% beef		Lost demand for sausage 70% chicken	
			Value	Percentage	Value	Percentage
1	180,000	300,000	16,900	9.4	29,400	9.8
2	360,000	600,000	60,571	16.8	105,777	17.6
3	540,000	900,000	120,100	22.2	215,489	23.9

#### 4.2.3. Analysis of sustainability dimensions

**Fig 4:**
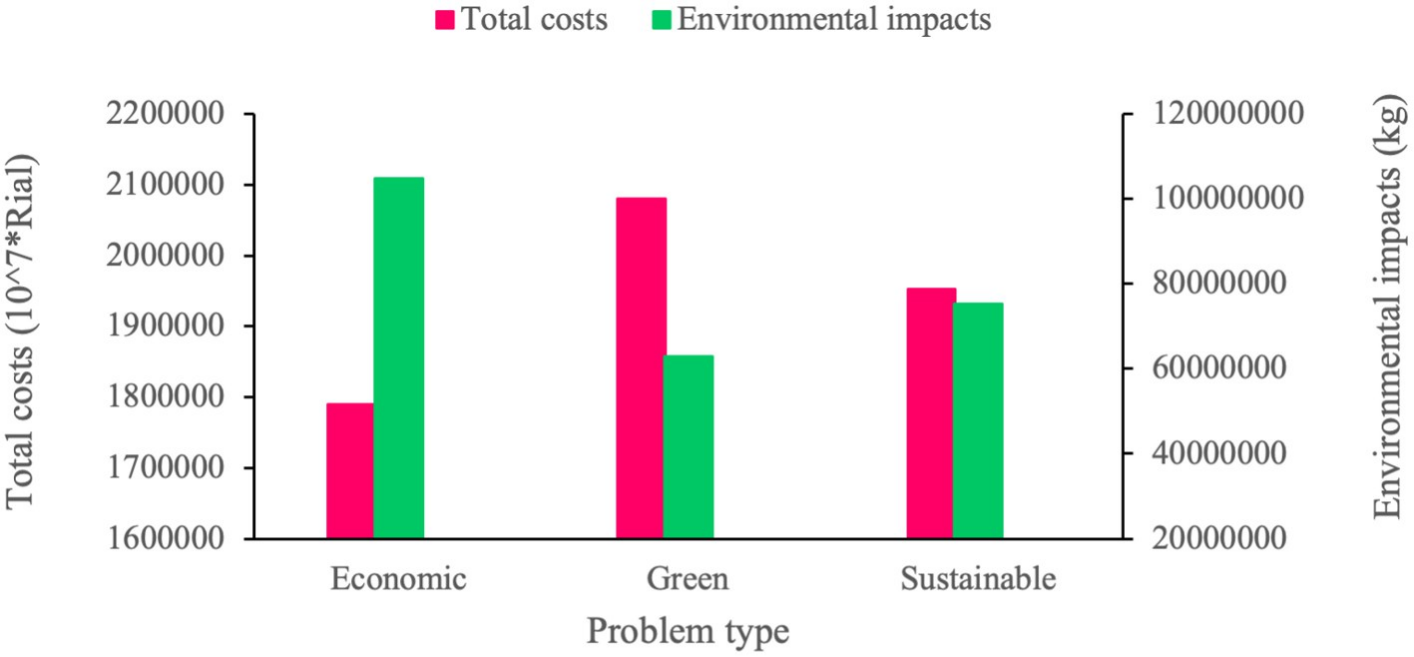
Economic and environmental objective functions sensitivity analysis on sustainability dimensions.

This section is dedicated to sensitivity analyses on the sustainability dimensions. For this purpose, we solve the proposed model in three different types: economic, green, and sustainable, and then proceed to discuss the results. In the economic type, only the economic objective function is addressed in the model; in the green type, both the economic and environmental objective functions are addressed simultaneously; and in the sustainable type, the problem is solved by considering the economic, environmental, and social objective functions, i.e. all three dimensions of sustainability. According to Table 21 and Fig 4, solving the problem in the economic type leads to the economic and environmental objective functions to take their best and worst values, respectively. However, focusing solely the economic dimension obtains the best value for the economic objective function. In the green type, by adding an environmental dimension to the problem, the opposite of the results of the economic type is obtained. That is, the economic and environmental objective functions take their worst and best values within the specified cases, respectively. Based on this observation, we may conclude that the green type increases the value of the economic objective in order to ensure a better value for the environmental objective. In the sustainable type, in order to establish a balance between the three dimensions of sustainability, economic and environmental objectives both gain values that lie between the values they were assigned in the economic and environmental types. Moreover, Table 19 indicates that the sustainable type results in a 9% increase in the total cost, as well as a 28.3% decrease in the environmental impacts, and a 53.6% increase in the social impacts compared to the economic type.

**Table 21:** Results of sensitivity analyses on sustainability dimensions.

Type of problem	Value of objective functions		
	Economic (×10^7^ Rials)	Environmental (kg)	Social
Economic	1,789,965	104,875,020	319.8
Green	2,080,660	62,954,850	422.2
Sustainable	1,952,306	75,179,800	491.4

#### 4.2.4. Analysis of the PFGP approach

**Fig 5:**
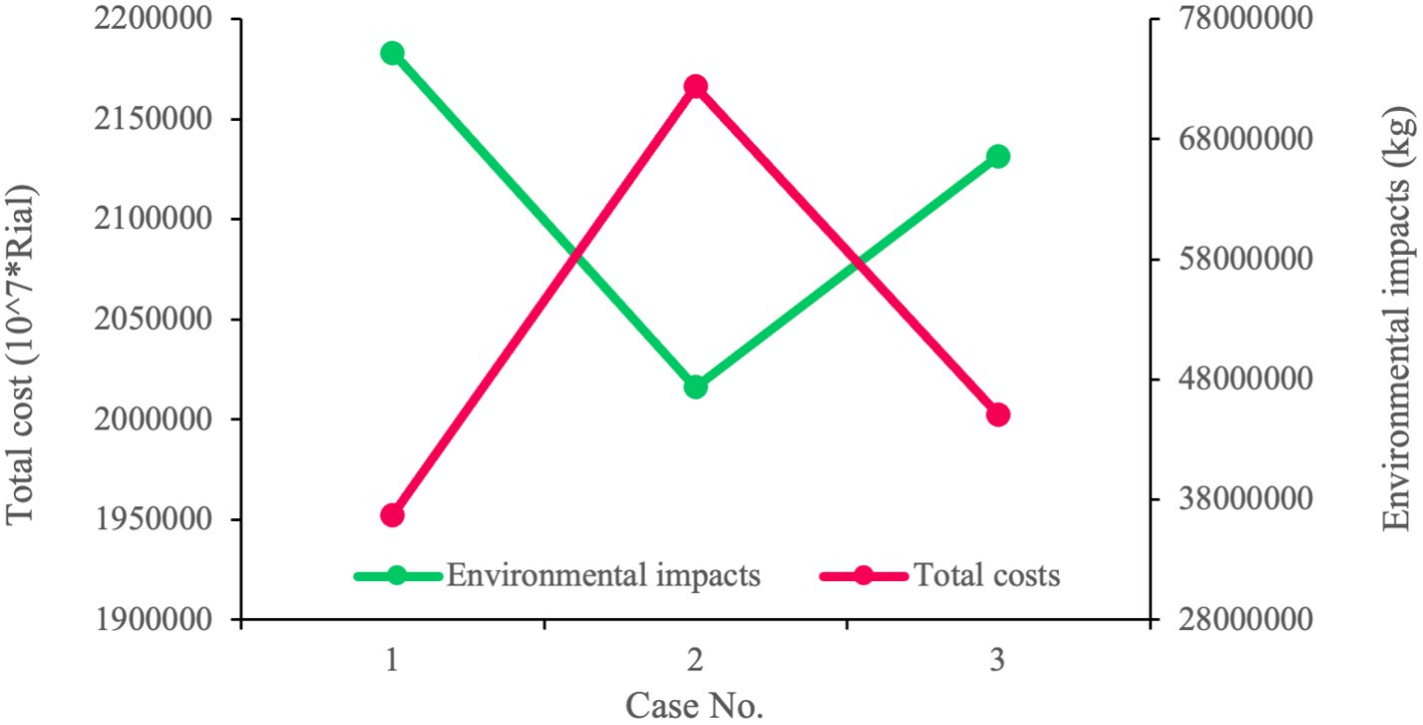
Conflict between cost and environmental impacts.

**Fig 6:**
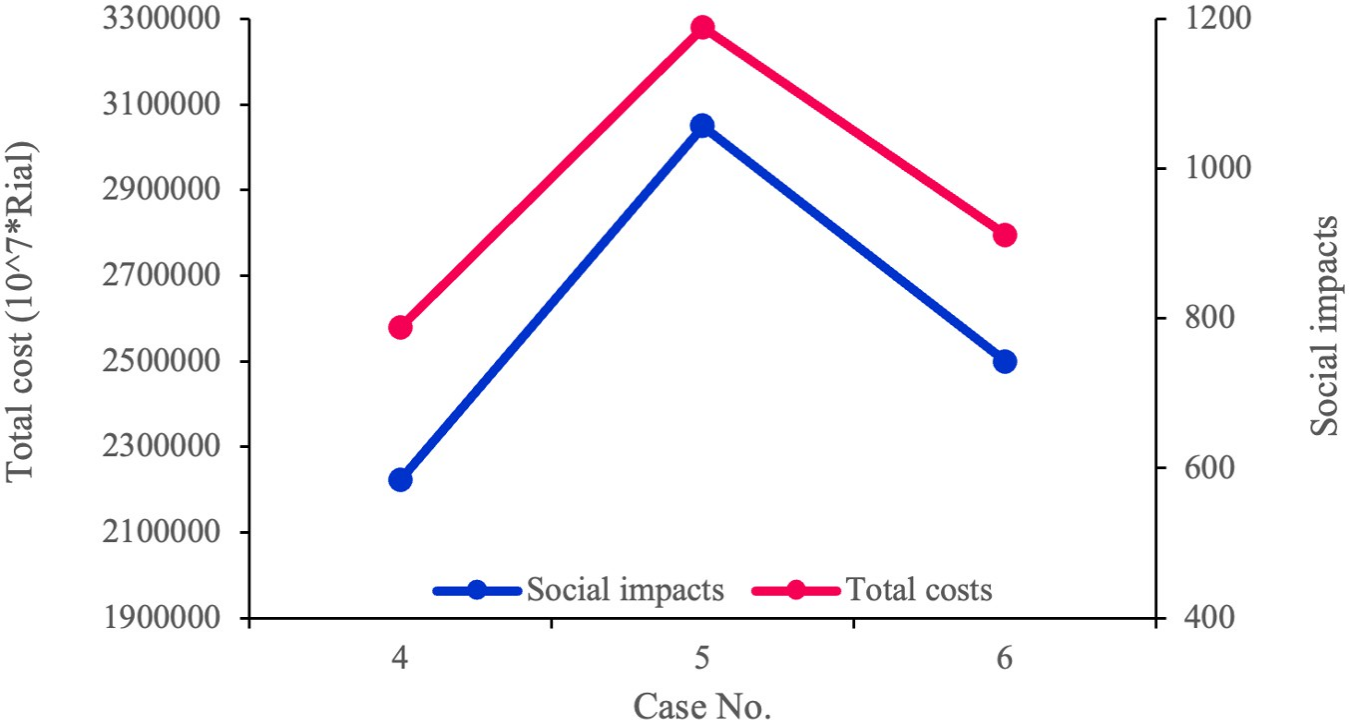
Conflict between cost and social impacts.

In the PFGP approach, the influential factor is the priority of objective functions, which is defined by their fuzzy satisfaction degrees (*λ*_*i*_). Therefore, we solve the model in six cases to assess the impact of changes in the objective functions’ priority on the problem. Table 20 shows the results of solving the model in the presented cases. Based on Table 20 and Fig 5, the conflicts between the economic and environmental goals are evident. In case 1, when the economic objective function takes the highest priority, it is assigned the best value, while the environmental objective function is assigned the worst. In Case 2, in which the priority of the second objective function is higher than the other two, the environmental impact is given the best value, with the economic objective function getting the worst. In case 3, where both the environmental and economic objectives are equally important, the value of both objective functions is squarely between their worst and best values. Table 22 and Fig 6 illustrate the conflict between the economic and social objective functions. In case 4, when the economic objective has the highest priority, it is assigned the best possible value. Conversely, the social objective function receives the worst value among all the cases. Based on case 5, when the social objective function becomes the top priority, it gets the best value, while the economic objective function has the worst value. Finally, in Case 6, in which the social and economic objectives share the top priority, the value of both objective functions is between their lowest and highest.

**Table 22:** Results of sensitivity analyses on the satisfaction degrees of the objective functions.

Case no.	Priority of objective functions	Value of objective functions		
		Economic (×10^7^ Rials)	Environmental (kg)	Social
1	*λ*_1_ > *λ*_2_ > *λ*_3_	1,952,306	75,179,800	491.4
2	*λ*_2_ > *λ*_1_ > *λ*_3_	2,166,354	47,356,670	537.8
3	*λ*_1_ = *λ*_2_ > *λ*_3_	2,002,396	66,592,640	529.2
4	*λ*_1_ > *λ*_3_ > *λ*_2_	2,579,849	83,160,790	584.6
5	*λ*_3_ > *λ*_1_ > *λ*_2_	3,279,308	103,202,400	1056.4
6	*λ*_1_ = *λ*_3_ > *λ*_2_	2,895,372	86,470,980	742.6

#### 4.2.5. Analysis of the risk-averse approach

**Fig 7:**
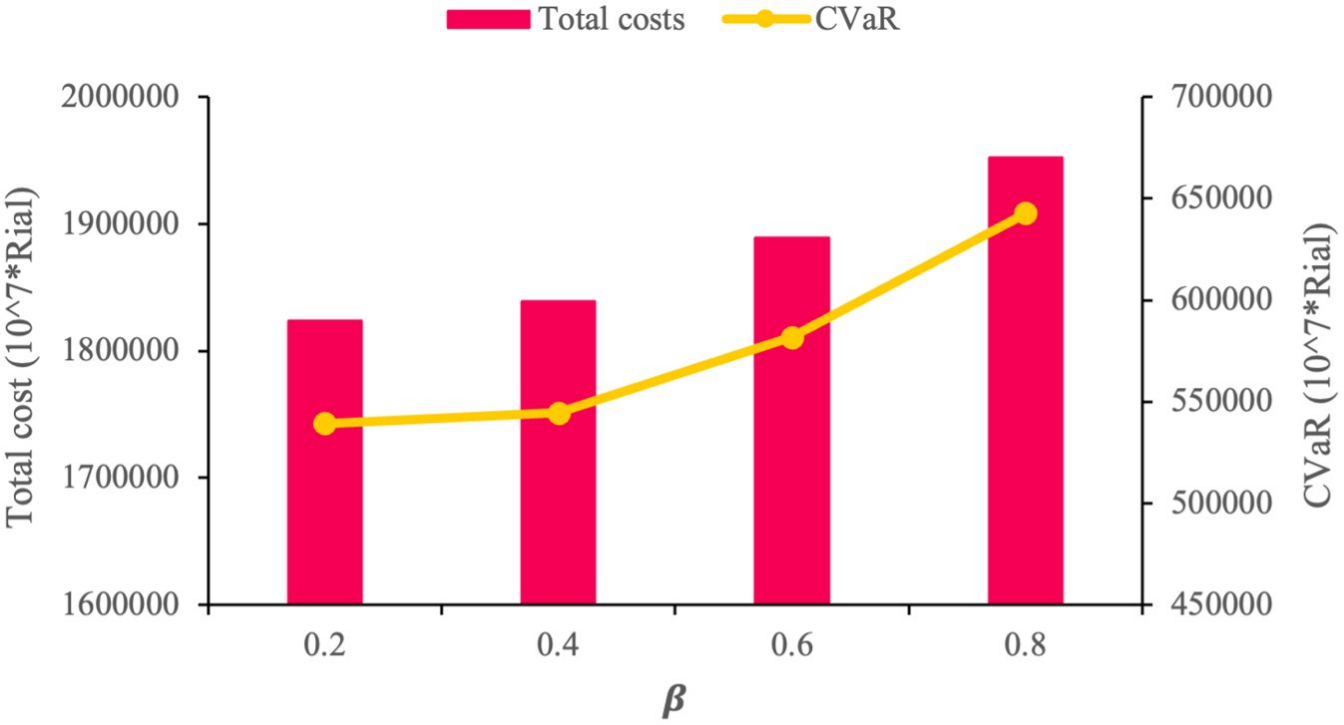
CVaR and total cost vs. *β*.

In this section, we assess the risk-aversion parameter in order to clarify how it may affect the objective functions of the proposed model and the obtained solutions. Consider an experiment with four different β values where the value of θ = 0.5 remains fixed to better comprehend how β impacts the model. Table 23 and Fig 7 show that as β grows larger, the value of the economic objective function increases, as well.

**Table 23:** Sensitivity of objective functions to the confidence level.

*θ*	*β*	Economic objective function (×10^7^ Rials)		Environmental objective function (kg)	Social objective function
		Total cost	CVaR		
0.5	0.2	1,823,513	539,415	62,592,390	542.2
0.5	0.4	1,839,075	544,650	62,970,050	542.2
0.5	0.6	1,888,650	581,639	64,868,610	509.4
0.5	0.8	1,952,306	642,698	75,179,800	491.4

**Fig 8:**
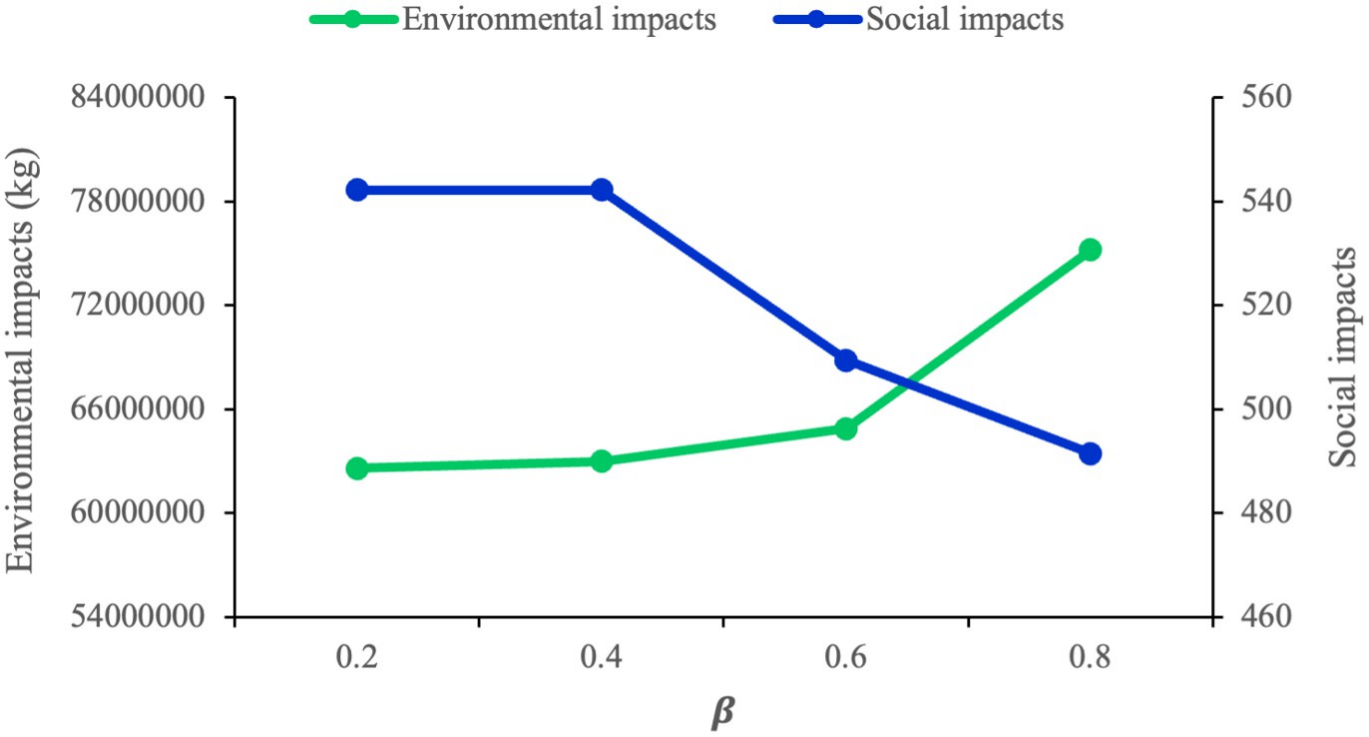
Environmental and social impacts vs. *β*.

Since CVaR measures the mean value of the worst (1 − *β*) % of the total expected cost, when the value of *β* increases, the risk aversion behavior of the proposed model improves. Hence, the model behaves more conservatively and the CVaR concentrates on the greater realization of total cost. Total costs are comprised of the first-stage costs, CVaR costs, and second-stage costs. It is also important to note that, the first- stage costs and consequently the number of established facilities do not change in response to varying risk parameters since they are not a function of CVaR. In other words, CVaR costs affect the second-stage costs. In general, we may draw the conclusion that a high risk-aversion strategy would result in higher levels of the second-stage costs and the total costs. To better comprehend this mechanism, we need to examine how *β* influences the environmental and social objective functions. As previously mentioned, by raising the value of *β* and giving the model a risk-averse attitude, the value of the economic objective function increases and the model should become more conservative, resulting in an upward trend in the value of the environmental impact and a downward trend in social impacts. This conflict can be clearly observed in Table 23 and Fig 8.

#### 4.2.6. Analysis of demand values

**Fig 9:**
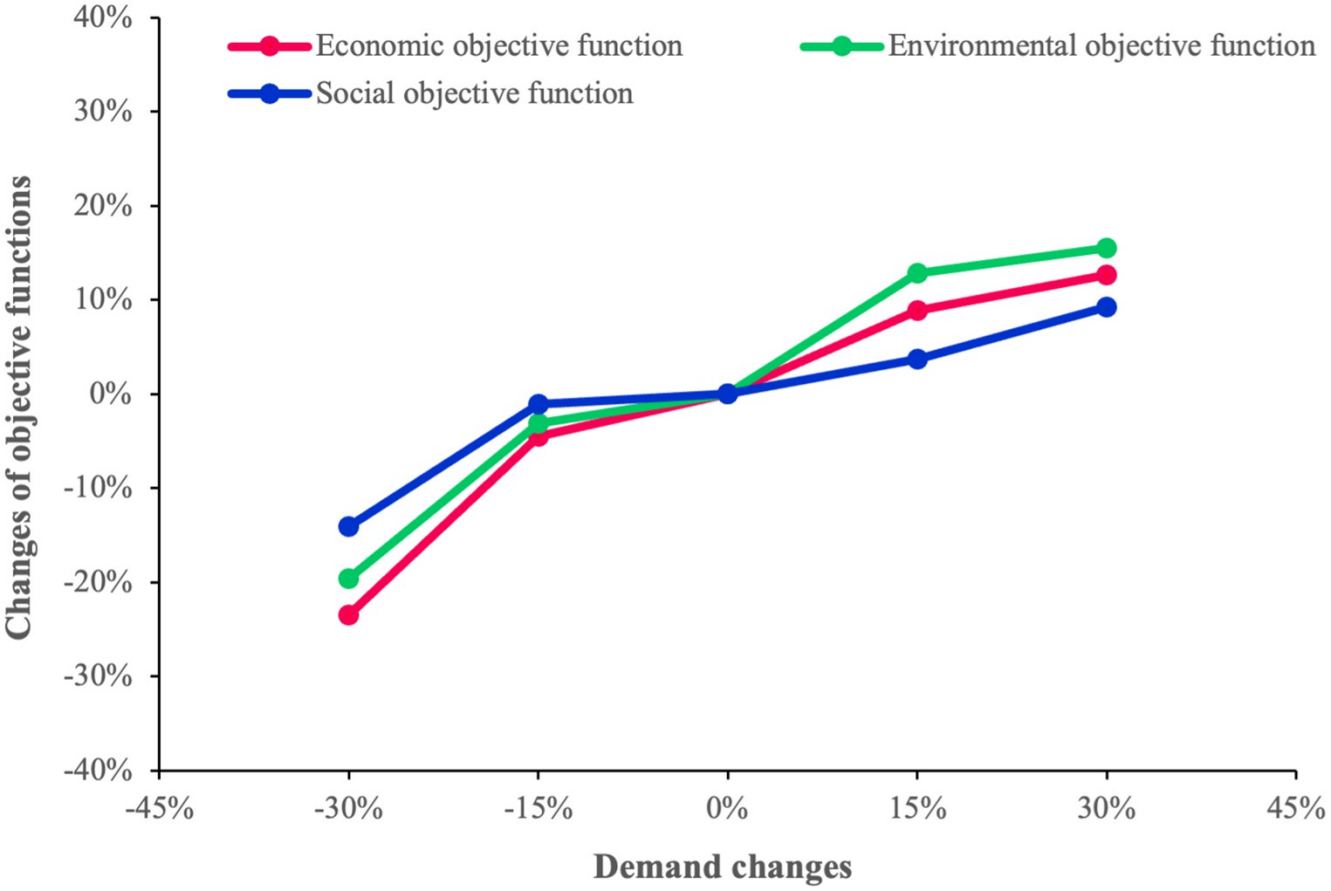
Behavior of the objective functions toward changes in demand.

To examine the impact of changes in demand on the value of the objective functions, we solve the model in five demand modes: -30%, -15%, 0%, +15%, and +30%). The results of the sensitivity analyses are shown in Fig 9. As expected, rising demand values increase the total SC costs. This may be a consequence of establishing new high-capacity facilities, increased raw material procurement and production of finished products, as well as transportation between the network nodes to satisfy the demand. Furthermore, as more high-capacity facilities are activated and transportation between facilities is raised, which leads to the use of higher capacity transportation modes, the adverse environmental impacts of the network increase. In addition, as the demand grows, the model tends to create more jobs by establishing more facilities and activating higher capacity levels to meet the retailers’ demand. In case of a significant increase in demand, the proposed model tries to keep the total SC costs at a minimum by not establishing new facilities. This is because the costs of opening new facilities to fully meet the demand outweigh the costs incurred by lost demands. Therefore, the model prefers to avoid establishing new facilities, activates lower capacity levels, and only partially satisfies the demand. This results in a less steep upward trend in total cost and social impact. As Fig 9 indicates, the reaction of the economic, environmental, and social objective functions in the proposed model to changes in demand under different modes complies with the above-mentioned explanations, indicating the proper logic of the model.

#### 4.2.7. Analysis of comparison between current and proposed SC conditions

**Fig 10:**
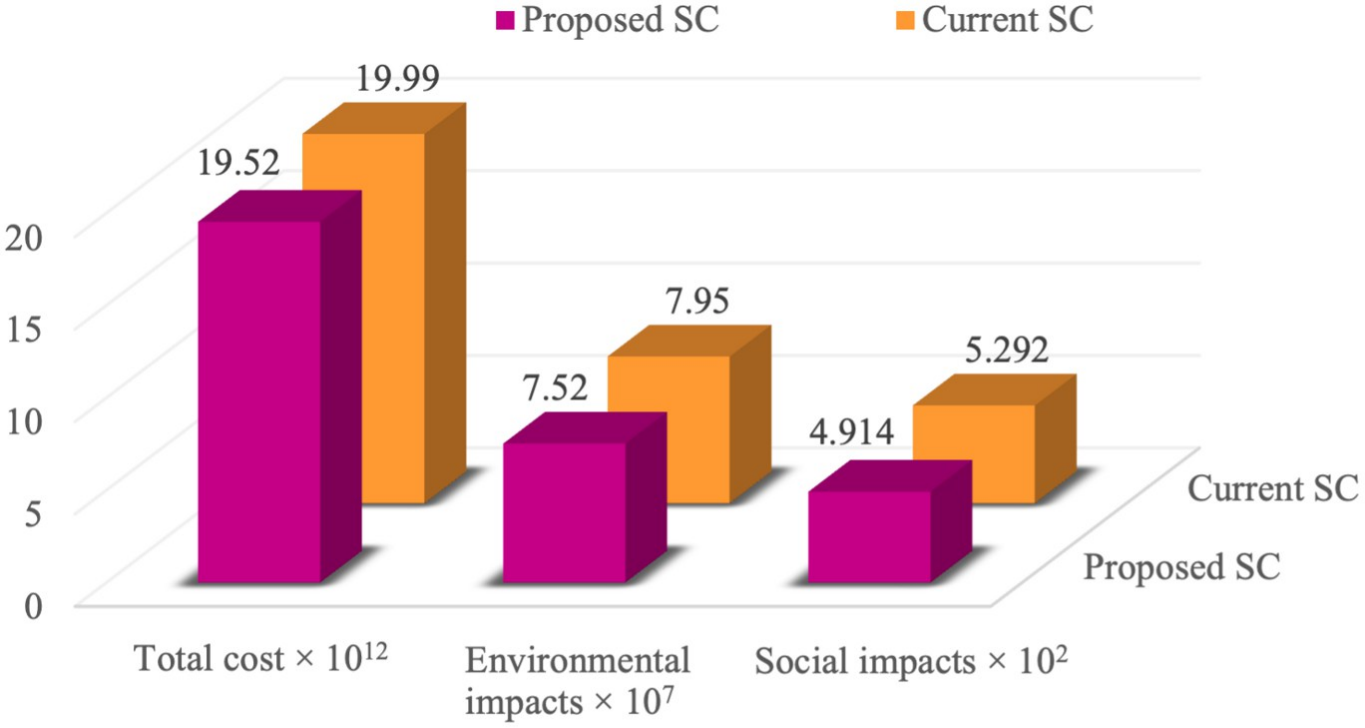
Comparison of current and proposed conditions of the case study.

Taking into account the current situation of the case study, which consists of one medium-capacity manufacturer and two high-capacity DCs, the values of the economic, environmental, and social objective functions are 1999168 × 10^7^, 79453400, and 529.2, respectively. In accordance with the opinions of the case study experts, the potential locations for construction of new facilities were selected based on the company’s revision plans regarding its supply chain to gain a larger market share. The results of solving the proposed model for the economic, environmental, and social objective functions are 1952306 × 10^7^, 75179800, and 491.4, respectively. Fig 10 compares the proposed and current conditions of the case study and displays the values of the model’s objective functions. As can be observed, compared to the current situation, the model proposed in this research causes a 2.3% drop in costs, a 5.4% drop in adverse environmental impacts, and a 7.1% drop in social impacts. This is achieved by constructing a medium-capacity manufacturers along with one medium-capacity and two low-capacity DCs. Thus, it can be said that the proposed model, whose candidate locations for the company’s future plans were selected by the experts, improves the current situation in terms of costs and environmental impacts. In addition, considering the high priority of the total SC costs, the downward trend of the social objective function in favor of the economic objective function seems logical.

### 4.3. Discussion

In this study, we developed a sustainable risk-averse model to design a SC for processed food products under uncertain conditions. To this end, a multi-period multi-objective model was presented to make decisions on facility location, optimal sourcing, transportation mode selection, capacity planning, and inventory management. The resulting model addresses three dimensions of sustainability as economic, environmental, and social objective functions, thereby minimizing the total cost and carbon emissions and maximizing the number of job opportunities created and safety of the FSC. The main contribution of this research to the literature may lie in our decision to incorporate a risk measure into the design of a sustainable FSC; an innovation that has never been attempted by previous researchers. Furthermore, we applied a scenario-based TSSP approach to cope with the uncertainty of demand for finished products and utilized a novel PFGP multi-objective method to solve the mathematical model. Also, we compared our proposed model with a situation in which the CvaR risk measure was not taken into account. Finally, results show that the value of cost function in risk-averse problem is 8.65% more than risk-neutral one. Because it behaves more conservatively and offers more resilient solutions in the presence of uncertainty compared to a risk-neutral problem.

## 5. Managerial insights and practical implications

Practitioners and academics can achieve significant outcomes by solving real-world case studies aligned with distinctive industry characteristics. Selecting the processed food industry in Iran and optimizing FSC, which contributes to all dimensions of sustainability, can be beneficial for food industry executives. Several useful managerial insights can be drawn from the analysis of the numerical results, including the following:

- The results indicate that lost demand is one of the negative consequences of the proposed FSC and appropriate policies should be implemented to manage this defect. As Table 18 indicates, the unmet demand dramatically increases when the demand for finished products increases because the model prefers to cope with periodic shortages rather than construct new facilities at enormous costs to fully meet the demand. In real-world conditions, however, SC managers can use a variety of strategies to decrease the unmet demand. An example is expanding the production capacity of manufacturers to prevent unmet demands. This extended capacity to produce more products, on the one hand, eliminates lost demands and, on the other hand, leads to new job opportunities created as social benefits of the SC. Another strategy for minimizing lost demand, which is especially useful in cases where the demand suddenly spikes is to schedule extra working shifts.
- The optimal configuration of the sustainable SC presented in this paper may provide decision-makers with practical solutions to manage the SC in terms of three dimensions of sustainability and their potential conflicts. As such, manufacturers can optimize their total cost, minimize the adverse environmental impacts of their operations, enhance their technologies, find new ways of ensuring staff satisfaction, and modify their reconstruction decisions to achieve an acceptable balance between the dimensions of sustainability. It is highly likely that this balance will have a positive effect on minimizing CO_2_ emissions, slowing down the alarming rate of climate change, boosting the local employment rate, and also tackling the human capital flight phenomenon currently plaguing many countries around the world by creating more job opportunities (see Table 19). Furthermore, as a practical strategy, government subsidies and incentives may be significantly effective in persuading employers and executives to overlook the continual rise of expenses in favor of upgrading the social and environmental circumstances of their respective businesses.
- Decision-makers can achieve higher levels of risk-aversion by increasing the values of *β*, causing the risk-averse model to behave more conservatively to generate better solutions in worst-case scenarios. However, as Table 21 and Fig 7 suggest, risk-aversion is often associated with increased total cost. It can perhaps be said that the more risk-averse the decision-makers are, the more importance they attach to risk parameters and, consequently, the model increases the total cost of the SC.

## 6. Conclusions and outlook

This research advances the field of FSC by addressing perishability in inventory decisions based on the production and delivery times of the products. A scenario-based TSSP approach that incorporates CvaR as a risk criteria into the objective function is employed to ensure a robust network in an uncertain environment. Finally, investigating a real case study in processed food industry, several strategic decisions including facility location, supplier selection, capacity planning, and transportation mode selection are made in the proposed FSCN. Based on sensitivity analyses on crucial parameters, findings of our research are as follows:

- We compared the proposed model (risk-averse problem) with risk-neutral one. Based on the findings, the network’s costs increased to 8.65% as the model’s conservativity grew, since it generated robust solutions in worst-case scenarios (see Fig 3).
- This research examined the service level of the SC in terms of lost demands. Results revealed that when the demand for the finished products increased by 50% in probable scenario, the quantities of lost demand for the two products increased by 32.1% and 35.7%, respectively. Thus, unmet demand is most likely caused by insufficient production capacity (see Table 18).
- Incorporating sustainability dimensions into the model and ignoring 9% of the total cost in favor of other objectives would lead to a 28.3% decrease in the adverse environmental impacts and a 53.6% increase in positive social impacts (see Table 19 and Fig 4).
- The outcome of the numerical analyses on the PFGP approach revealed that the final values of objective functions might vary according to changes in their priority based on decision-makers’ preferences (see Table 20 and Figs 5 and 6).
- We adjusted CVaR confidence level with four distinct values to account for sensitivity analysis. According to the results, a higher CVaR confidence level and a risk-averse attitude would increase the economic objective function value, and make the model more conservative (see Table 21 and Fig 7).
- The results indicated that growing the demand values would increase the total SC costs. It could be due to the establishment of new high-capacity facilities, increased raw material procurement and production of finished products, as well as transportation between the network nodes to meet the growing demand (see Fig 9).
- The comparison between current and proposed SC conditions showed that the model presented in this research results in a 2.3% decrease in costs, a 5.4% decrease in detrimental environmental impacts, and a 7.1% decrease in social impacts (see Fig 10).

The limitations of the research are obtaining optimal solutions on a large scale and within an acceptable timeframe. To address this issue, we recommend applying exact and evolutionary algorithms in combination with novel heuristic and meta-heuristic algorithms, such as those put forward by [63–65].

Finally, the authors would like to recommend the following as potentially promising subjects for further investigation. It is possible to experiment with different quality levels for raw materials and suppliers can place various purchase discounts based on the quality and quantity of the raw materials required by manufacturers. Additionally, exploring global SC issues and factors may be a highly relevant research route, as most leading SCs in various sectors operate globally today. Furthermore, incorporating other risk criteria, such as Entropic VaR (EVaR) and Robust CvaR (RCVaR) may provide significant benefits for conservative decision-makers as future research, aiming to enhance the robustness of the model’s performance and enable it to effectively handle fluctuations [66]. Utilizing alternative methods, like stochastic, fuzzy, and robust optimization techniques that incorporate data-driven uncertain parameters can offer risk-averse decision-makers a more accurate representation of the situation, enabling them to make optimum decisions while reducing risks [64, 66, 67].
